# MUC17 is an essential small intestinal glycocalyx component that is disrupted in Crohn’s disease

**DOI:** 10.1172/jci.insight.181481

**Published:** 2024-12-19

**Authors:** Elena Layunta, Sofia Jäverfelt, Fleur C. van de Koolwijk, Molly Sivertsson, Brendan Dolan, Liisa Arike, Sara I.M. Thulin, Bruce A. Vallance, Thaher Pelaseyed

**Affiliations:** 1 Department of Medical Biochemistry and Cell Biology, Institute of Biomedicine, University of Gothenburg, Gothenburg, Sweden.; 2 Division of Gastroenterology, Hepatology and Nutrition, Department of Pediatrics, BC Children’s Hospital Research Institute, University of British Columbia, Vancouver, Canada.

**Keywords:** Cell biology, Gastroenterology, Cellular immune response, Glycobiology, Inflammatory bowel disease

## Abstract

Crohn’s disease (CD) is the chronic inflammation of the terminal ileum and colon triggered by a dysregulated immune response to bacteria, but insights into specific molecular perturbations at the critical bacteria-epithelium interface are limited. Here, we report that the membrane mucin MUC17 protected small intestinal enterocytes against commensal and pathogenic bacteria. In noninflamed CD ileum, reduced MUC17 levels and a compromised glycocalyx barrier allowed recurrent bacterial contact with enterocytes. *Muc17* deletion in mice rendered the small intestine particularly prone to atypical bacterial infection while maintaining resistance to colitis. The loss of Muc17 resulted in spontaneous deterioration of epithelial homeostasis and in the extraintestinal translocation of bacteria. Finally, Muc17-deficient mice harbored specific small intestinal bacterial taxa observed in patients with CD. Our findings highlight MUC17 as an essential region-specific line of defense in the small intestine with relevance for early epithelial defects in CD.

## Abstract 2

Patients with ileal Crohn’s disease carry diminished levels of membrane mucin MUC17. A preclinical mouse model demonstrates that MUC17 protects the small intestine against bacteria.

## Graphical abstract

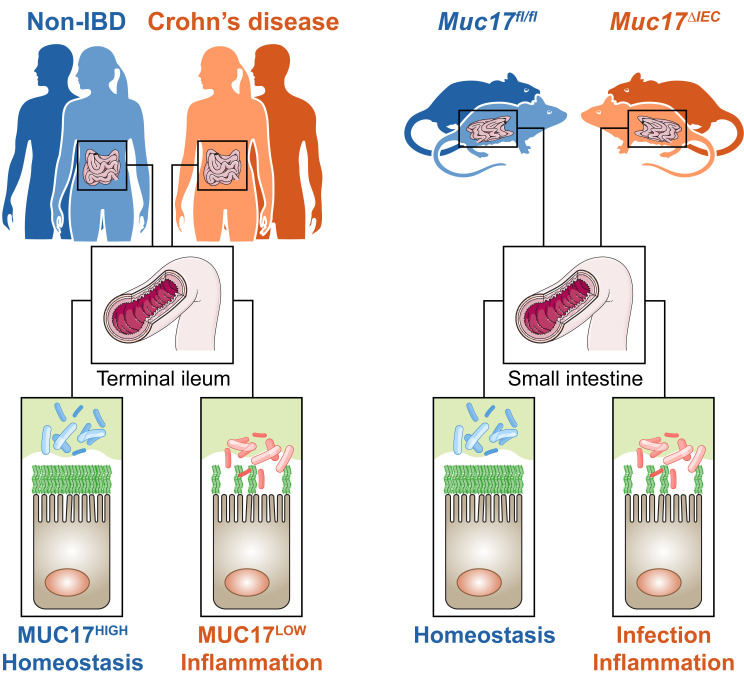

## Introduction

**Figure 1:**
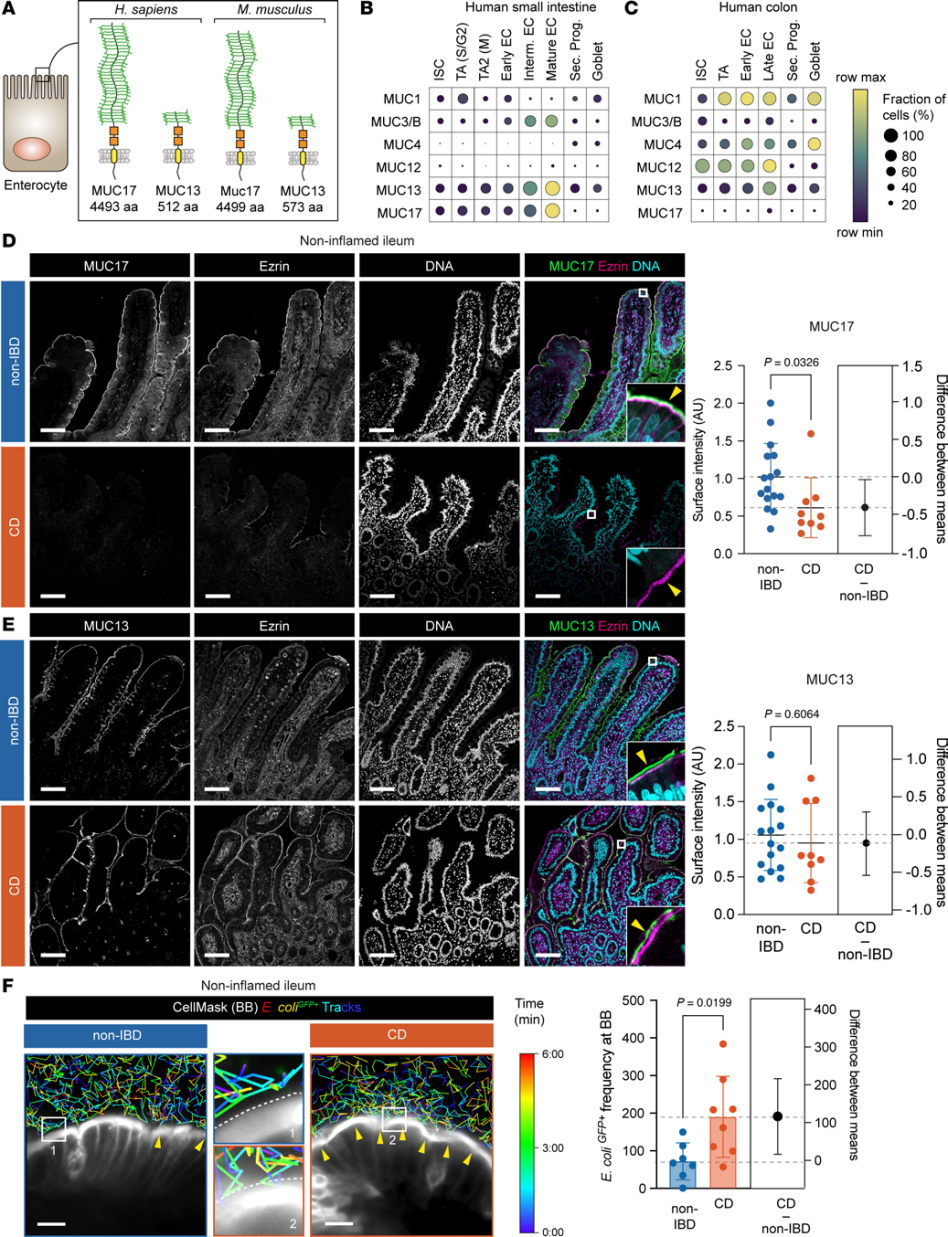
Reduced surface levels of MUC17 and a permeable glycocalyx in the noninflamed ileum of patients with Crohn’s disease. (**A**) Cartoon of human and mouse MUC17 and MUC13. (**B**) Membrane mucin mRNA transcripts at single-cell resolution in human small intestinal epithelial cells. (**C**) Membrane mucin mRNA transcripts in human colonic epithelial cells. Row maximum and minimum represent relative expression of each gene among cell types. EC, enterocyte; ISC, intestinal stem cell; TA, transit amplifying; Sec. Prog., secretory progenitor; Goblet, goblet cell; S/G2, S and G2 phase; M, M phase. (**D**) Immunohistochemistry of MUC17 (green), Ezrin (magenta), and DNA (cyan) in sections of noninflamed ileal biopsies from non-IBD and CD patients, alongside semiquantitative analysis of MUC17 levels in the brush border. Each channel is shown in grayscale. Yellow arrow points to the brush border. Scale bar, 100 μm. Insets, 30 µm. *n* = 16 for non-IBD, *n* = 9 for CD. (**E**) Immunohistochemistry of MUC13 (green), Ezrin (magenta), and DNA (cyan) in noninflamed ileal biopsies from non-IBD control and CD patients, alongside semiquantitative analysis of MUC13 in the brush border. Each channel is shown in grayscale. Yellow arrow points to the brush border. Scale bar, 100 μm. Insets, 30 µm. *n* = 16 for non-IBD, *n* = 9 for CD. (**F**) Time-lapse glycocalyx permeability assay in noninflamed ileal biopsies from non-IBD and CD patients, stained with CellMask and incubated with *E*. *coli^GFP+^* tracked over the course of 6 minutes. Yellow arrows show overlap between *E*. *coli^GFP+^* and CellMask. Dashed white line marks the brush border. Magnifications are labeled with numbers. Scale bar, 10 μm. Insets, 8 µm. Pseudocolor scale indicates time points during tracking bacteria. Bar graph depicts frequency of *E*. *coli^GFP+^* attached to the brush border. *n* = 7 for non-IBD, *n* = 8 for CD. Data are means ± SD. Significance was determined by unpaired 2-tailed *t* test (**D**–**F**). Estimation plot shows the difference between CD and non-IBD means with a 95% confidence interval.

Mucins are critical for the gastrointestinal defense system (1, 2). In the mouse distal colon, the gel-forming Muc2 forms an attached impenetrable mucus layer that separates bacteria from the epithelium (3). Defects in core mucus components constitute early steps in the pathogenesis of ulcerative colitis, a form of inflammatory bowel disease (IBD) in humans (4). In contrast, the mucus of the small intestine is unattached and permeable to luminal material, including bacteria (5). This region-specific difference demands a distinct defense mechanism to manage host-microbiota interactions. In this context, the role of membrane mucins expressed by small intestinal enterocytes is unexplored because of limited insight into their gene sequences (6) and difficulties preserving them in fixed tissues. MUC17, alongside MUC13, is a conserved heavily *O*-glycosylated membrane mucin positioned at the apical brush border of differentiated enterocytes (7, 8) (Figure 1A). In the mouse small intestine, the homeostatic cytokine IL-22 induces Muc17 expression and the establishment of a surface glycocalyx that in ex vivo experiments prevents direct attachment of bacteria to the brush border (9).

Crohn’s disease (CD) is an IBD subtype characterized by patchy chronic inflammation involving dysregulated immune responses to the gut bacteria (10). Unlike ulcerative colitis that is limited to the colon, CD affects both the small and large intestines, with almost one-third of patients displaying inflammation exclusively in the terminal ileum (11). Susceptibility to ileal CD has been linked to mutations in the intracellular peptidoglycan sensor *NOD2* and autophagy protein *ATG16L1*, which impair the detection and degradation of invasive bacteria that cross the apical cell membrane (12–14). However, apart from reports of transcriptional changes in enterocyte brush border components (15), it is still unclear whether patients with CD have any functional protein defects at the apical membrane, upstream of the intracellular innate immune response to bacteria. MUC17 is both transcriptionally and structurally associated with the brush border (9), but the role of MUC17 in epithelial defense and whether MUC17 expression and glycocalyx integrity are compromised in CD are not known.

Here, we analyzed noninflamed ileal biopsies from patients with CD and non-IBD controls together with a genetic mouse model to define the function of MUC17 in the intestine. Patients with CD displayed decreased MUC17 levels and a glycocalyx that was more permeable to bacteria. In mice, intestine-specific deletion of the *Muc17* gene was dispensable for colonic inflammation while rendering the small intestine abnormally sensitive to infection by the attaching effacing bacterium *Citrobacter rodentium* (*C*. *rodentium*). *Muc17^ΔIEC^* mice also suffered spontaneous deterioration of the epithelial cell barrier and displayed translocation of viable bacteria to extraintestinal tissues. Last, we showed that Muc17 regulates the abundance of specific bacterial taxa in the small intestine, including bacteria enriched in patients with CD. Thus, the *Muc17^ΔIEC^* model reproduces fundamental manifestations of CD, suggesting that MUC17 is essential for protecting the small intestine against commensal and pathogenic bacteria.

## Results

### Decreased MUC17 levels and glycocalyx integrity in ileal CD.

In humans, MUC17 is exclusively expressed in differentiated enterocytes of the small intestine (Figure 1, B and C). We analyzed the expression of MUC17 in histological sections from the noninflamed terminal ileum of patients with CD and a control group with non-IBD-related pathologies (Supplemental Table 1; supplemental material available online with this article; https://doi.org/10.1172/jci.insight.181481DS1). MUC17 levels were significantly reduced in the enterocyte brush border of the CD compared with non-IBD ileum, which showed a typical MUC17 staining in the brush border marked by Ezrin (Figure 1D). Membrane mucin MUC13 was not altered between the 2 cohorts (Figure 1E). Next, we analyzed ileal biopsies with an ex vivo time-lapse glycocalyx permeability assay (9), which measures the ability of the glycocalyx to separate GFP-expressing *Escherichia coli* (*E*. *coli^GFP+^*) from the brush border (see Methods). In line with decreased apical MUC17 in CD ileum, the frequency of *E*. *coli^GFP+^* in contact with the brush border was significantly higher in CD compared with non-IBD controls, indicating reduced glycocalyx barrier integrity in patients with CD (Figure 1F, Supplemental Figure 1A, and Supplemental Video 1). Subsequent staining of biopsies analyzed by glycocalyx permeability assay for MUC17 surface staining showed that non-IBD samples had a significantly higher relative MUC17 surface expression than CD samples (1.26 ± 0.51 versus 0.33 ± 0.07) (Supplemental Figure 1, B and C). Thus, the noninflamed CD ileum exhibits an impaired glycocalyx barrier and reduced MUC17 levels in the apical brush border.

### The Muc17^ΔIEC^ mouse lacks a small intestinal glycocalyx.

**Figure 2:**
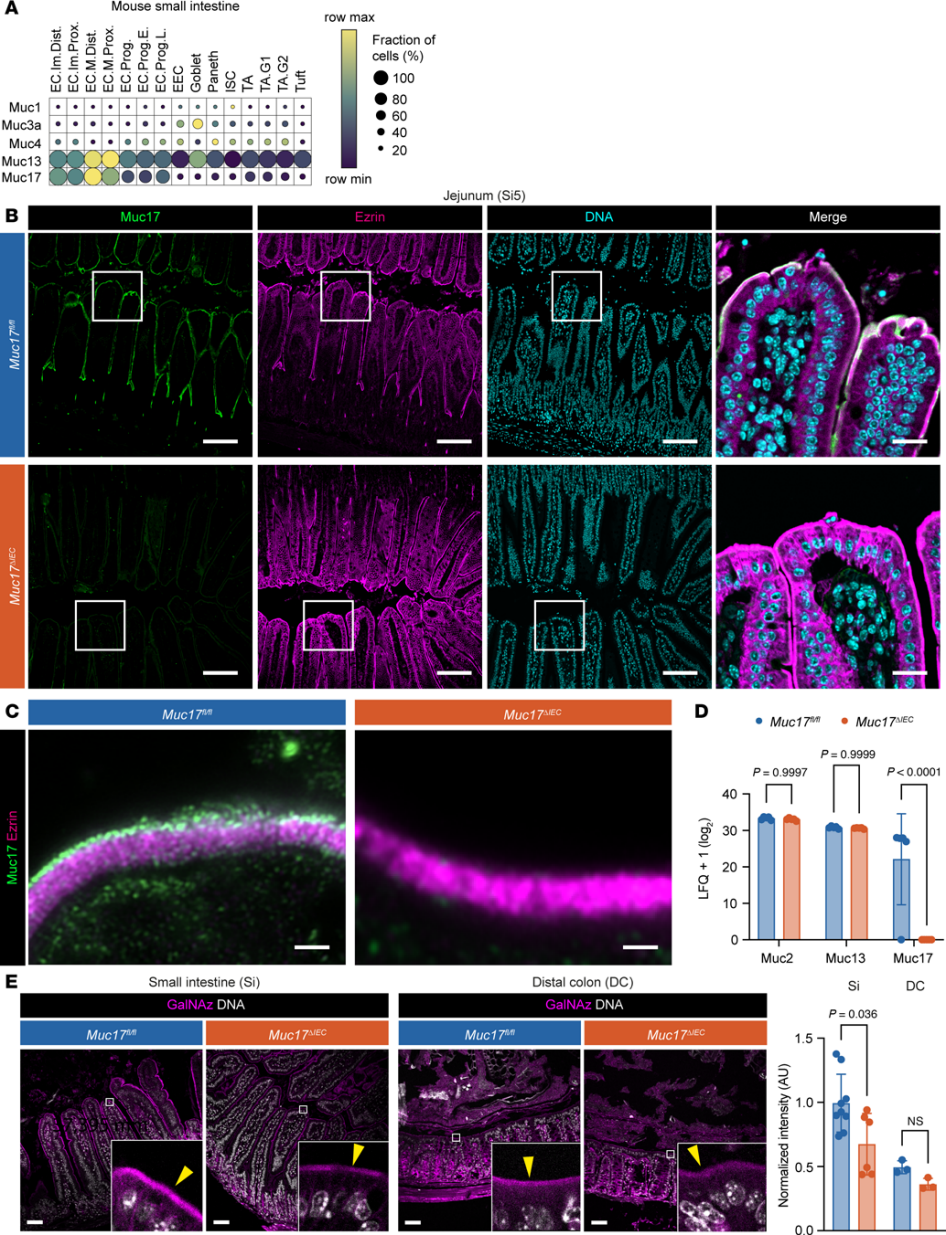
Epithelial cell–specific deletion of *Muc17* results in the loss of Muc17 expression in the mouse small intestine. (**A**) Expression of membrane mucin transcripts in epithelial cell types of the mouse small intestine. The color scheme shows row maximum and minimum representing the relative expression of each gene among all cell types. EC, enterocyte; EEC, enteroendocrine cell; ISC, intestinal stem cell; TA, transit amplifying; Im, immature; M, mature; Prox, proximal; Dist, distal; Prog, progenitor; G1, G1 phase; G2, G2 phase. (**B**) Immunohistochemistry of Muc17 (green), Ezrin (magenta), and DNA (cyan) in histological sections of the jejunum (Si5) from *Muc17^fl/fl^* and *Muc17*^ΔIEC^ mice. Scale bar 50 μm. Scale bar in insets 10 μm. (**C**) Airyscan high-resolution microscopy of Muc17 (green) and Ezrin (magenta) in the small intestinal brush border of *Muc17^fl/fl^* and *Muc17*^ΔIEC^ mice. Scale bar 1 μm. (**D**) Bar graph representing the abundance of detected mucins in the proteome of small intestinal epithelial cells from *Muc17^fl/fl^* and *Muc17*^ΔIEC^ mice. *n* = 5 for each group. (**E**) Representative confocal micrographs of small intestine (Si) and distal colon (DC) from *Muc17^fl/fl^* and *Muc17*^ΔIEC^ mice, stained for bio-orthogonally labeled *O*-glycans (azide derivative of *N*-acetylgalactosamine, GalNAz; magenta) and DNA (white). Scale bar 50 μm. Bar graphs represent the quantification of GalNAz intensity in the apical brush border of the Si and DC in each group. Si5: *n* = 6–9 per group. DC: *n* = 3 per group. Significance was determined by 2-way ANOVA followed by Holm-Šidák correction (**D**) and Mann-Whitney test (**E**).

The loss of glycocalyx integrity in CD ileum suggests that the loss of apical MUC17 is an early defect resulting in increased bacterial contact with the brush border. To test this hypothesis, we generated a conditional *Muc17^ΔIEC^* mouse by crossing *Muc17^fl/fl^* and *Vil1-Cre* mice expressing the Cre recombinase in intestinal epithelial cells (Supplemental Figure 2, A–C). Unlike human MUC17, murine Muc17 is expressed in both the small intestine and colon (Figure 1A; Figure 2, A and B; and Supplemental Figures 3 and 4). The successful deletion of *Muc17* was thus verified in the duodenum, jejunum (Si5), ileum (Si8), proximal colon, and distal colon (DC) (Figure 2B and Supplemental Figures 3 and 4). High-resolution microscopy of small intestinal enterocytes revealed that Muc17 was lacking from the tips of microvilli in *Muc17^ΔIEC^* mice (Figure 2C). *Muc17* deletion was also demonstrated in microvillus-derived luminal vesicles isolated from the small intestine (16, 17) (Supplemental Figure 2D). To evaluate the impact of *Muc17* deletion on other mucins, we performed proteomic analysis of isolated small intestinal epithelial cells from *Muc17^fl/fl^* and *Muc17^ΔIEC^* cohoused littermates. Deletion of *Muc17* did not result in a compensatory change in the abundance of Muc13 or the gel-forming Muc2 (Figure 2D and Supplemental Table 2). Moreover, *Muc17^ΔIEC^* mice did not exhibit alterations in tissue morphology of the small intestine or DC (Supplemental Figure 2E). Notably, the deletion of *Muc17* had no impact on brush border morphology or cellular polarity, evaluated by the brush border markers Ezrin and Cdhr5 and by basolateral EpCAM (Supplemental Figure 2, F and G). Mucin *O*-glycans convert the glycocalyx into a size-selective diffusion barrier and shield against bacterial degradation (1, 9). To determine if the loss of Muc17 affected the density of glycocalyx *O*-glycans, we employed in vivo bio-orthogonal labeling using GalNAz, the azide derivative of *N*-acetylgalactosamine, the first glycan added to serines or threonines during mucin-type *O*-glycosylation (18). GalNAz intensity was significantly reduced in the small intestinal brush border of *Muc17^ΔIEC^* mice compared with controls but remained unchanged in the colonic brush border (Figure 2E). The *O*-glycan deficit of the small intestinal glycocalyx was further supported by decreased staining with Aleuria aurantia lectin and wheat germ agglutinin (WGA), which bind fucose and *N*-acetylglucosamine, respectively (Supplemental Figure 2H). Differential protein expression analysis did not detect any significant changes in glycosyltransferases involved in the initiation, branching, or capping of mucin-type *O*-glycans (Supplemental Figure 2I and Supplemental Table 2), indicating that reduced GalNAz incorporation in the brush border of *Muc17^ΔIEC^* mice was a result of the absence of Muc17 and not an altered glycosylation machinery. Thus, under baseline conditions, *Muc17* deletion results in an explicit loss of the mucin-based glycocalyx without impacting the overall tissue and cell morphology of the small intestine.

### Deletion of Muc17 causes abnormal small intestinal susceptibility to C. rodentium infection.

**Figure 3:**
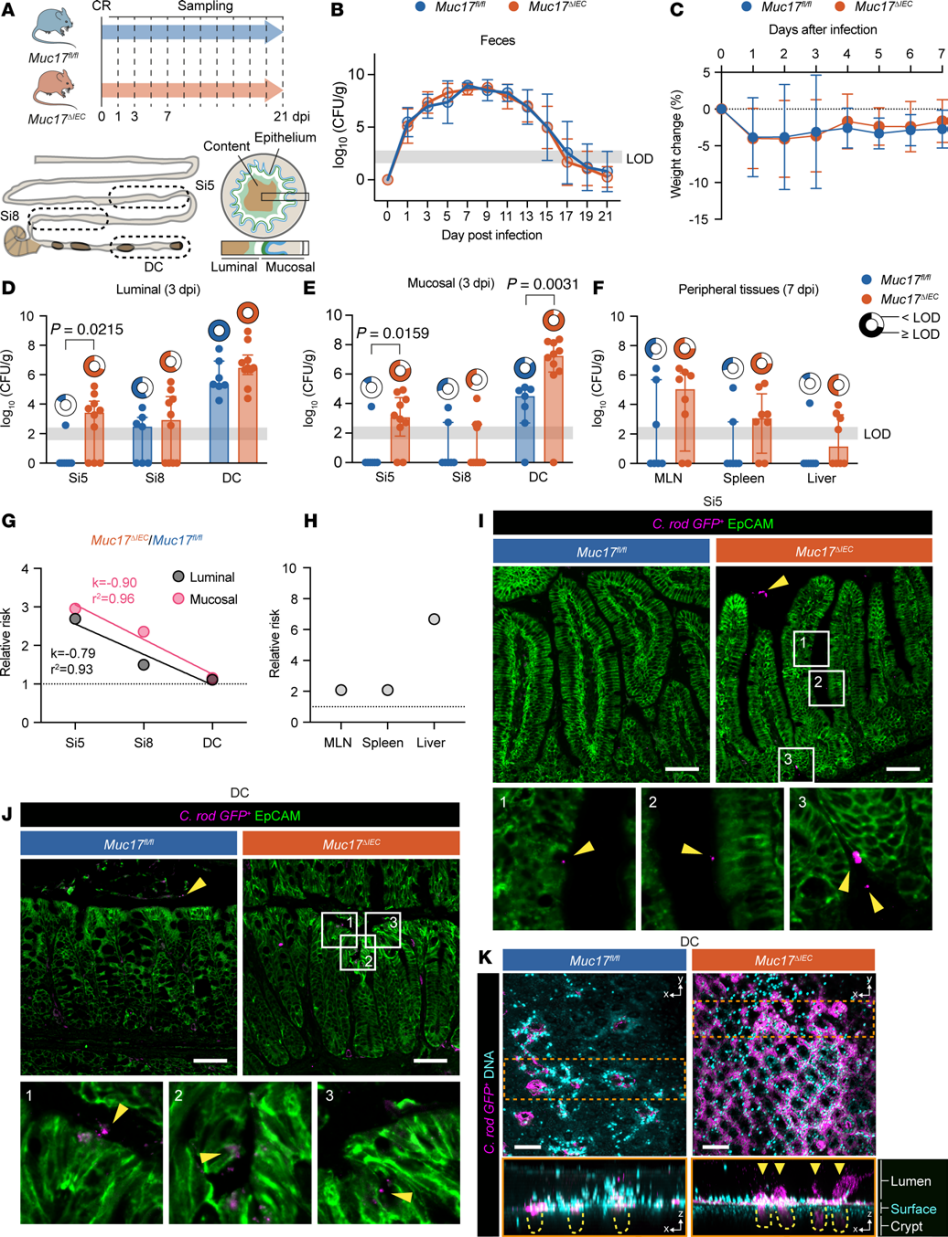
Deletion of Muc17 results in abnormal small intestinal susceptibility to *C*. *rodentium* infection. (**A**) Infection protocol and sampling time points. Si5, jejunum; Si8, ileum; DC, distal colon. (**B**) *C*. *rodentium* CFU in fecal samples at various days postinfection (dpi). (**C**) Weight loss of infected *Muc17^fl/fl^* and *Muc17*^ΔIEC^ mice as percentage of the initial weight at 0 dpi. (**D**) *C*. *rodentium* CFU in luminal compartments of Si5, Si8, and DC at 3 dpi. (**E**) *C*. *rodentium* CFU in mucosal compartments of Si5, Si8, and DC at 3 dpi. (**F**) *C*. *rodentium* CFU in mesenteric lymph nodes (MLNs), spleen, and liver at 7 dpi. Circle charts represent proportion of mice with CFU above the limit of detection (LOD) in each segment or tissue site. (**G**) Relative risk of carrying *C*. *rodentium* CFU above LOD in Si5, Si8, and DC luminal and mucosal compartments. *n* = 6–10 per group. (**H**) Relative risk of carrying *C*. *rodentium* CFU above LOD in MLNs, spleen, and liver. *n* = 6–10 per group. (**I**) Immunohistochemistry of *C*. *rodentium^GFP+^* (magenta) in relation to epithelium (EpCAM, green) in the Si5 of *Muc17^fl/fl^* and *Muc17*^ΔIEC^ mice 3 dpi. Yellow arrows point to bacterial cells. Scale bar, 50 μm. (**J**) Immunohistochemistry of *C*. *rodentium^GFP+^* (magenta) in relation to epithelium (EpCAM, green) in the DC of *Muc17^fl/fl^* and *Muc17*^ΔIEC^ mice 3 dpi. Yellow arrows point to bacterial cells. Scale bar, 50 μm. (**K**) Visualization of *C*. *rodentium^GFP+^* (magenta) in relation to epithelium (DNA, cyan) in explants of *Muc17^fl/fl^* and *Muc17*^ΔIEC^ DC 3 dpi. Upper panels show top view (*x*,*y* plane) and lower panels show extended orthogonal view (*x*,*z* plane) of boxed region (orange). Penetration of *C*. *rodentium^GFP+^* (magenta) into colonic crypts (yellow dashed lines) is highlighted (yellow arrows). Scale bar, 100 μm. Significance was determined by Mann-Whitney test (**D**–**F**).

Since we did not observe any baseline phenotype in *Muc17^ΔIEC^* mice, cohoused *Muc17^fl/fl^* and *Muc17^ΔIEC^* littermates were infected with *C*. *rodentium*, a natural murine pathogen that normally causes a self-limiting infection in the distal colon (19). Infection kinetics in each group were monitored over a period of 21 days postinfection (dpi) (Figure 3A). Regardless of genotype, fecal *C*. *rodentium* counts peaked at 7–9 dpi and declined below the limit of detection (LOD) by 17 dpi (Figure 3B). Both groups exhibited a slight but comparable weight loss during the initial infection phase (1–3 dpi) (Figure 3C). Next, we quantified *C*. *rodentium* colonization in the luminal and mucosal compartments of the jejunum (Si5), ileum (Si8), and DC at 3 dpi. As expected, *Muc17^fl/fl^* mice exhibited a robust colonization in the DC (Figure 3, D and E). In *Muc17^ΔIEC^* Si5, however, we observed 3 orders of magnitude higher *C*. *rodentium* counts compared with the control group (Figure 3, D and E). The abnormal *C*. *rodentium* infection of Si5 was not limited to the luminal compartment but was also detected in the mucosa, indicating that the small intestinal epithelium was more susceptible to *C*. *rodentium* infection in the absence of Muc17 and a glycocalyx. Greater pathogen counts were also observed in the mucosa of *Muc17^ΔIEC^* DC compared with the infected controls (Figure 3E). There was also a trend toward increased translocation of *C*. *rodentium* to the peripheral tissues in *Muc17^ΔIEC^* mice at 7 dpi (Figure 3F). Notably, across all time points after infection, a larger fraction of *Muc17^ΔIEC^* mice carried a detectible pathogen burden in the small intestine compared with *Muc17^fl/fl^* mice (Supplemental Figure 5). At 7 dpi, 91% ± 6% of all *Muc17^ΔIEC^* mice exhibited a small intestinal infection compared with only 54% ± 13% of the *Muc17^fl/fl^* mice. A similar analysis of colonic pathogen burden did not reveal any significant difference between the groups. Accordingly, the relative risk of *C*. *rodentium* infection in *Muc17^ΔIEC^* mice peaked in Si5 mucosa and decreased toward the DC, suggesting that Muc17 primarily serves as a small intestinal defense mechanism during bacterial infection (Figure 3G). *Muc17* deletion also resulted in a higher relative risk of pathogen translocation to the peripheral tissues (Figure 3H). Histological analysis 3 dpi revealed numerous *C*. *rodentium^GFP+^* cells in contact with the epithelium at villus apexes, intervillar regions, and the crypt compartments of *Muc17^ΔIEC^* Si5, whereas *C*. *rodentium^GFP+^* was not detected in *Muc17^fl/fl^* Si5 (Figure 3I). Assessment of the DC showed *C*. *rodentium^GFP+^* at the surface epithelium and in the crypts of *Muc17^ΔIEC^* mice (Figure 3, J and K). Collectively, the absence of Muc17 caused an abnormal small intestinal susceptibility to *C*. *rodentium*, indicating that Muc17 is essential for protecting the small intestinal epithelium against pathogenic bacteria.

### Muc17^ΔIEC^ mice display spontaneous loss of epithelial homeostasis.

**Figure 4:**
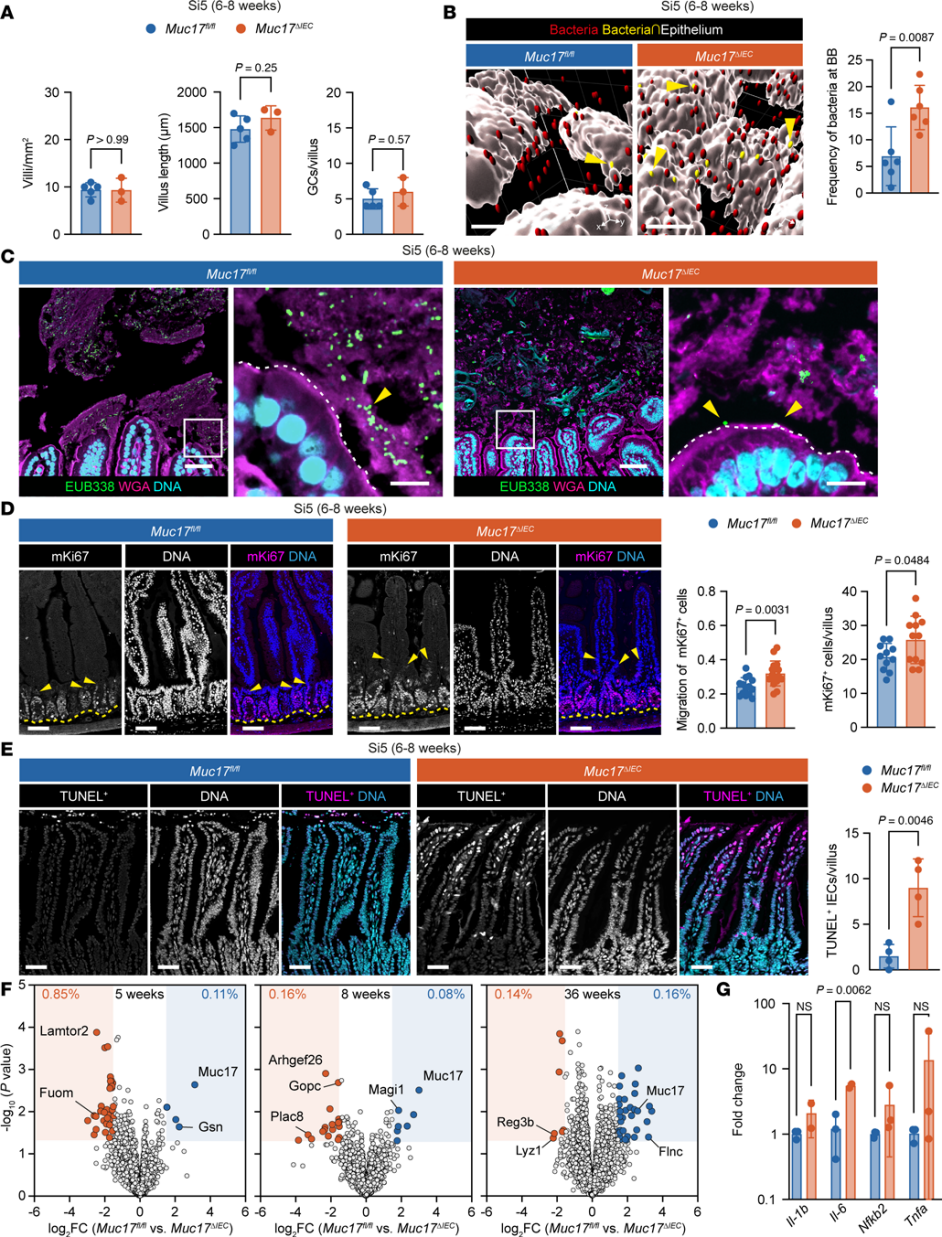
Epithelial barrier dysfunction in *Muc17^ΔIEC^* small intestine under SPF conditions. (**A**) Quantification of villi/mm^2^ of histological section, villus length, and GCs per villus in *Muc17^fl/fl^* and *Muc17*^ΔIEC^ jejunum (Si5). *n* = 5 for *Muc17^fl/fl^*; *n* = 3 for *Muc17*^ΔIEC^. (**B**) Visualization of commensal bacteria (red) on Si5 epithelium (white). Bacteria in contact with epithelium are shown in yellow. Scale bar, 100 μm. Quantification shows frequency of bacteria attached to the brush border in each cohort. *n* = 6 for *Muc17^fl/fl^*; *n* = 6 for *Muc17*^ΔIEC^. (**C**) *Muc17^fl/fl^* and *Muc17*^ΔIEC^ Si5 stained for bacteria (EUB338, green), epithelium and mucus (WGA, magenta), and DNA (cyan). Yellow arrows point to bacteria. Scale bar 25 μm. Scale bar in insets 10 μm. (**D**) Immunohistochemistry of mKi67 (magenta) and DNA (blue) in *Muc17^fl/fl^* and *Muc17*^ΔIEC^ Si5. Each channel is shown in grayscale. Yellow arrows point to mKi67^+^ cells with maximum migration along villus-crypt axis. Yellow line depicts the crypt bottom. Scale bar, 50 μm. Quantification of mKi67^+^ cell migration along crypt-villus axis and absolute numbers of mKi67^+^ per villus in *Muc17^fl/fl^* and *Muc17*^ΔIEC^ Si5. *n* = 3–6 villi per mouse, 3 mice per group. (**E**) Immunohistochemistry of TUNEL^+^ nuclei (magenta) and DNA (cyan) in *Muc17^fl/fl^* and *Muc17*^ΔIEC^ Si5. Each channel is shown in grayscale. Scale bar 50 μm. Quantification of TUNEL^+^ cells per villus in *Muc17^fl/fl^* and *Muc17*^ΔIEC^ mice. *n* = 4 for *Muc17^fl/fl^*; *n* = 4 for *Muc17*^ΔIEC^. (**F**) Volcano plots showing protein abundance in epithelial cells of Si5 from 5-, 8-, and 36-week-old *Muc17^fl/fl^* compared with *Muc17*^ΔIEC^ mice. Differentially expressed proteins (fold-change ≥ 2, *P* value < 0.05) are highlighted with filled circles. *n* = 5 for each genotype in each age group. (**G**) Expression of *Il-1b*, *Il-6*, *Nfkb2*, and *Tnfa* genes in the Si5 of *Muc17^fl/fl^* and *Muc17*^ΔIEC^ mice. Significance was determined by Mann-Whitney test (**A**) and unpaired 2-tailed *t* test (**B**, **D**, **E**, and **G**).

Given that Muc17 protected the small intestine against pathogenic infection, we reasoned that *Muc17* deletion might also allow commensal bacteria to make direct contact with enterocytes and thereby disrupt epithelial homeostasis. Thus, we turned our attention to unchallenged 6- to 8-week-old cohoused *Muc17^fl/fl^* and *Muc17^ΔIEC^* littermates maintained under standard pathogen–free (SPF) conditions. Histological analysis of Si5 did not reveal any genotype-dependent differences in crypt number, villus length, or goblet cell frequency (Figure 4A and Supplemental Figure 2E). Next, Si5 explants were flushed, then fixed to preserve the mucosal compartment, and the distribution of bacteria in relation to the brush border was assessed using confocal microscopy and automatic image segmentation. Compared with *Muc17^fl/fl^*, significantly higher numbers of bacteria were directly bound to the brush border of *Muc17^ΔIEC^* mice (Figure 4B). Findings were supported by fluorescence in situ hybridization (FISH) with a universal eubacterial probe, showing bacteria in direct contact with the *Muc17^ΔIEC^* brush border (Figure 4C). Uncontrolled bacterial interactions with the epithelium can trigger increased epithelial proliferation and cell death (20, 21). Correspondingly, we observed a significant expansion of proliferative mKi67^+^ epithelial cells within the crypt compartment of *Muc17^ΔIEC^* Si5 (Figure 4D). Free nuclear DNA 3′-OH termini generated by single-strand breaks are hallmarks of apoptosis. Compared with controls, *Muc17^ΔIEC^* Si5 displayed a higher number of TUNEL-positive apoptotic cells at villus apexes (Figure 4E). Next, we used mass spectrometry to evaluate the impact of *Muc17* deletion on epithelial cell homeostasis. Label-free protein quantification in epithelial cells from 5-, 8-, and 36-week-old *Muc17^fl/fl^* and *Muc17^ΔIEC^* mice revealed altered protein abundance in 0.25%–1.00% of the proteome, mainly affecting brush border–associated proteins (Figure 4F and Supplemental Table 2). Specifically, we observed a higher abundance of Rho guanine nucleotide exchange factor 26 (Arhgef26) in epithelial cells of 8-week-old *Muc17^ΔIEC^* mice. Arhgef26 participates in enterocyte membrane remodeling and ruffling during bacterial invasion (22) (Figure 4F and Supplemental Figure 6). We also detected higher abundance of the antimicrobial proteins Lyz1 and Reg3b in 36-week-old *Muc17^ΔIEC^* mice (Figure 4F and Supplemental Figure 6), reflecting a host response to persistent bacterial challenges exerted on the intestinal epithelium in *Muc17^ΔIEC^* mice. Further assessment showed no histological signs of inflammation (Supplemental Figure 7) but a significant upregulation of the proinflammatory cytokine *Il-6* in the small intestine of *Muc17^ΔIEC^* mice compared with wild-type littermates (Figure 4G). Collectively, our results suggest that the absence of Muc17 leads to a higher burden of bacteria at the epithelial surface, which results in the spontaneous loss of epithelial homeostasis and inflammation.

### Muc17 is dispensable for protection against chemically induced colitis.

To define the protective role of Muc17 in the murine distal colon, cohoused *Muc17^fl/fl^* and *Muc17^ΔIEC^* littermates were subjected to 7-day ad libitum administration of dextran sodium sulfate (DSS), a chemically induced model of human ulcerative colitis (Supplemental Figure 8A). *Muc17^ΔIEC^* mice displayed a reduction in weight compared with wild-type mice but only at the end of the intervention (day 6) (Supplemental Figure 8B). While there was a significant shortening of the colon in *Muc17^ΔIEC^* mice after 7 days (Supplemental Figure 8C), only a moderately increased susceptibility to DSS was measured by stool consistency, fecal blood score, overall disease activity index, and survival (Supplemental Figure 8, D–G). Our observations were supported by histological analysis of the Si5 and DC, which corroborated that *Muc17^fl/fl^* and *Muc17^ΔIEC^* mice responded similarly to DSS-induced colitis (Supplemental Figure 8, H and I). The late onset of moderate inflammation in the absence of Muc17 contrasted acute induction of severe colitis in *C3GnT^–/–^*, *Vamp8^–/–^*, and *Tgm3^–/–^* mice with defects in Muc2 glycosylation, secretion, and cross-linking (23–25). Thus, we concluded that Muc17 does not play a critical protective role in the DC.

Further assessment of *Muc17^ΔIEC^* mice under baseline conditions did not show any deviations in the number of crypts, crypt length, or goblet cell frequency in the DC (Supplemental Figure 9A and Supplemental Figure 2E). Next, we quantified the barrier properties of the inner mucus layer (IML) separating bacteria from the epithelium (26). FISH showed a defined separation of bacteria from the epithelium in 6- to 8-week-old *Muc17^fl/fl^* and *Muc17^ΔIEC^* mice (Supplemental Figure 9B). Ex vivo mucus penetrability analysis in viable explants from 6- to 8- or 36- to 40-week-old mice, evaluating the penetration of microbeads through the IML, showed that both *Muc17^ΔIEC^* and *Muc17^fl/fl^* mice maintained an impenetrable IML typically observed in wild-type mice (3) (Supplemental Figure 9, C and D). *Muc17^ΔIEC^* mice did, however, display elevated occurrence of shed cells (Supplemental Figure 9D) and increased epithelial cell proliferation measured by mKi67^+^ cells (Supplemental Figure 9E). The fact that *Muc17^ΔIEC^* mice were susceptible to small intestinal infection by a colonic pathogen strongly suggests that Muc17 is a critical, but probably not the only, defense mechanism that protects the small intestine against pathogens. That *Muc17^ΔIEC^* mice are only slightly more susceptible to acute DSS challenge, a model for colonic inflammation (colitis), suggests that Muc17 does not play a critical role in the colonic defense system. This is logical because the impenetrable IML, which constitutes the main defense barrier of the colon, remains intact in the absence of Muc17 (Supplemental Figure 9, C and D). Together, our findings verify that Muc17 is not required for the protection of the DC against commensal bacteria.

### Muc17 deletion alters the small intestinal microbiota and elicits extraintestinal translocation of bacteria.

**Figure 5:**
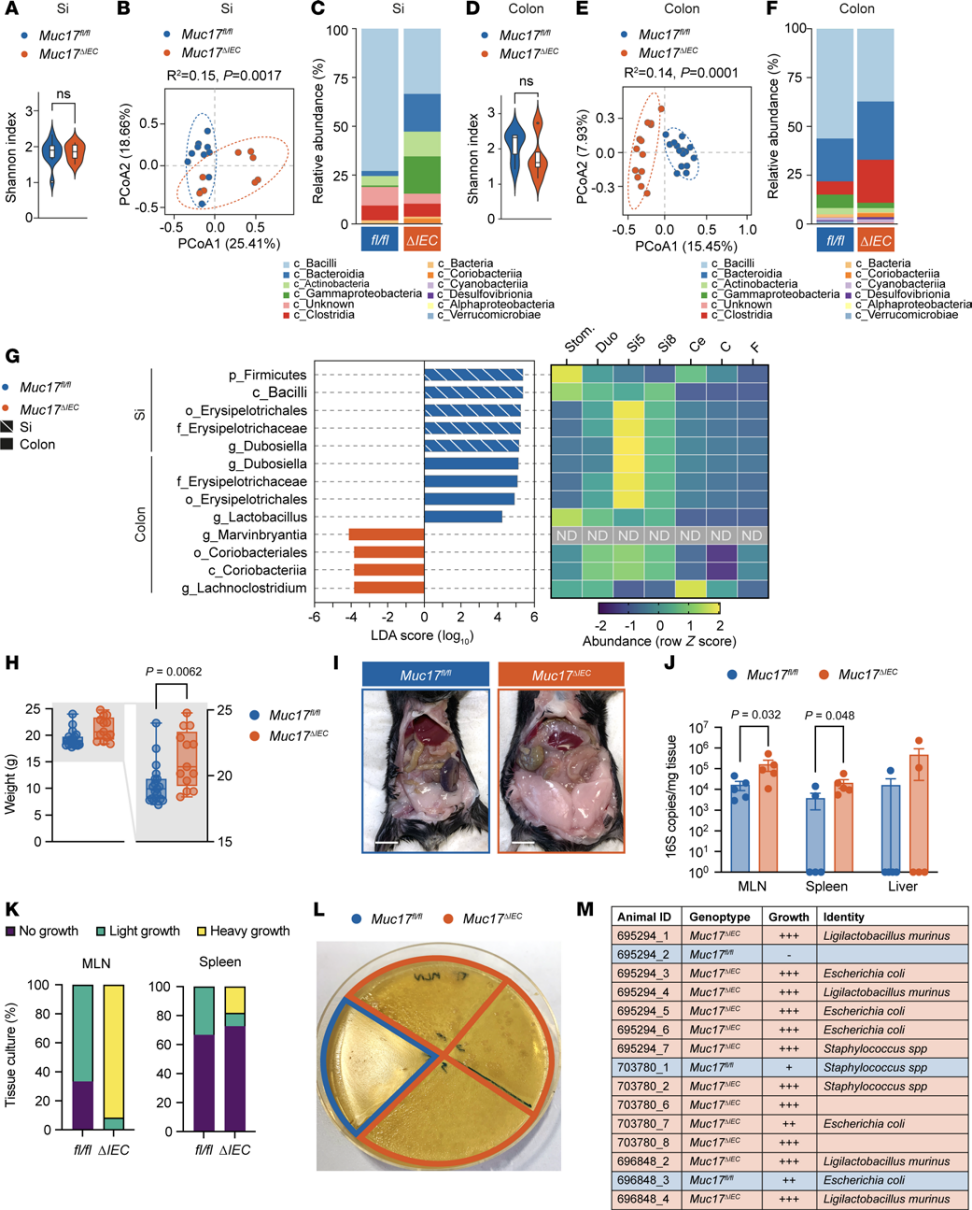
Deletion of Muc17 results in alterations in luminal microbiota composition and translocation of commensal bacteria to peripheral tissues. (**A**) The α-diversity (Shannon index) of luminal bacterial communities in the Si of *Muc17^fl/fl^* (*n* = 11) and *Muc17*^ΔIEC^ (*n* = 8) mice. (**B**) Principal coordinate analysis of β-diversity (Bray-Curtis dissimilarity) of luminal bacterial communities of *Muc17^fl/fl^* and *Muc17*^ΔIEC^ Si. (**C**) Relative frequency of major classes found in *Muc17^fl/fl^* and *Muc17*^ΔIEC^ Si. (**D**) The α-diversity (Shannon index) of luminal bacterial communities in the colon of *Muc17^fl/fl^* (*n* = 15) and *Muc17*^ΔIEC^ (*n* = 13) mice. (**E**) Principal coordinate analysis of β-diversity (Bray-Curtis dissimilarity) of luminal bacterial communities in *Muc17^fl/fl^* and *Muc17*^ΔIEC^ colon. (**F**) Relative frequency of major classes found in *Muc17^fl/fl^* and *Muc17*^ΔIEC^ colon. (**G**) Linear discriminant analysis (LDA) effect size identification of small intestinal and colonic taxa that differentiate between *Muc17^fl/fl^* (blue) and *Muc17*^ΔIEC^ (orange) mice. The heatmap shows the row *z*-score representing the relative abundance of the selected taxa in different gastrointestinal segments. Stom, stomach; Duo, duodenum; Si5, jejunum; Si8, ileum; Ce, cecum; C, colon; F, feces; ND, not detected. (**H**) Total weight of cohoused *Muc17^fl/fl^* (*n* = 19) and *Muc17*^ΔIEC^ (*n* = 13) littermate mice. Box plots show the median and minimum to maximum values for each group. (**I**) Representative image of the opened abdomen of 36- to 40-week-old *Muc17^fl/fl^* and *Muc17*^ΔIEC^ mice. Scale bar, 1 cm. (**J**) Quantification of bacterial 16S copy number in MLNs, spleen, and liver tissue by quantitative PCR. *n* = 5 for each group. (**K**) Quantification of bacterial growth in tissue homogenates of MLNs and spleen on BHIS agar plates under anaerobic conditions. (**L**) Representative image of bacterial growth in tissue homogenates of MLNs on BHIS agar under anaerobic conditions. (**M**) Identification of isolated bacterial colonies identified by 16S rRNA sequencing. Significance was determined by unpaired 2-tailed *t* test (**A**) and Mann-Whitney test (**C**).

*Muc17* deletion resulted in susceptibility to *C*. *rodentium* and increased contact between commensal bacteria and small intestinal enterocytes, but the precise effect of Muc17 on the region-specific composition of the microbiota is not understood. We thus analyzed bacterial genomic DNA from the luminal compartment of the small intestine and colon of cohoused *Muc17^fl/fl^* and *Muc17^ΔIEC^* littermates by 16S rRNA gene sequencing. While there was no significant difference in species richness and evenness (α-diversity) between the 2 groups (Figure 5, A and D), the β-diversity based on Bray-Curtis distances revealed distinct patterns in the microbiota of *Muc17^fl/fl^* and *Muc17^ΔIEC^* mice (Figure 5, B and E). The small intestinal community of *Muc17^ΔIEC^* mice was characterized by a higher abundance of the classes of Actinobacteria, Bacteroidia, Coriobacteria, and gammaproteobacteria (Figure 5C), while the colonic microbiota shifted toward Clostridia, Coriobacteria, and Desulfovibrionia (Figure 5F). Linear discriminant analysis effect size analysis showed a depletion of Firmicutes, Bacilli, and the genus *Dubosiella*, belonging to Erysipelotrichales, from the *Muc17^ΔIEC^* small intestine (Figure 5G and Supplemental Figure 10A). The small intestinal alterations in bacterial composition were largely reproduced in the *Muc17^fl/fl^* colon, with the enrichment of Erysipelotrichales and *Dubosiella* alongside *Lactobacillus* (Figure 5G and Supplemental Figure 10B). In addition, Coriobacteriales, *Lachnoclostridium*, and *Marvinbryantia* were enriched in the *Muc17^ΔIEC^* colon (Figure 5G and Supplemental Figure 10B). Combined, the affected genera comprised an average of 15%–20% of the detected operational taxonomic units. Since the microbiota composition varies along the intestine (27), we assessed the regional distribution of the differentially enriched bacterial taxa. Bacilli, Coriobacteria, and Erysipelotrichales had a primarily small intestinal niche, with Erysipelotrichales dominating the jejunum (Si5) (Figure 5G), thus suggesting a transmission of compositional changes from the small intestine to the colon. Collectively, *Muc17* deletion induced a substantial shift in the abundance of specific bacteria that colonize the small intestine (Supplemental Figure 10C), suggesting that Muc17 regulates the region-specific selection of commensal bacteria.

In ileal CD, displacement of mucosa-associated bacteria from the site of inflammation to mesenteric adipose tissue induces the expansion of encapsulating “creeping fat” (28). Bacterially induced enlargement of visceral adipose tissue also occurs in mice that fail to control microbial translocation (29). Under baseline SPF conditions, *Muc17^ΔIEC^* mice displayed a higher total body mass (Figure 5H) and larger abdominal fat pads compared with age-matched *Muc17^fl/fl^* cohoused littermates (Figure 5I). Subsequently, we asked if *Muc17^ΔIEC^* mice displayed translocation of commensal bacteria to extraintestinal tissues. Absolute 16S rRNA gene quantification uncovered significantly higher counts in the mesenteric lymph nodes (MLNs) and spleen of *Muc17^ΔIEC^* mice compared with wild-type mice (Figure 5J). Bacterial quantification was complemented with anaerobic culturing of viable bacteria from the affected tissues. Heavy bacterial growth was observed in 92% of *Muc17^ΔIEC^* MLNs, with the remaining 8% exhibiting light growth (Figure 5, K and L). In *Muc17^fl/fl^* mice, 33% of MLNs were clear of bacteria and 67% exhibited only light growth. We also observed a higher number of *Muc17^ΔIEC^* spleens with heavy bacterial growth compared with control tissues (Figure 5K). Sequencing of the 16S gene identified the translocated bacteria as *Escherichia* *coli*, *Staphylococcus* spp., and *Ligilactobacillus* *murinus* (Figure 5M), the latter previously described as a translocating strain (30). Thus, we concluded that viable commensal bacteria penetrate the intestinal epithelial cell barrier in the absence of Muc17, highlighting its key role in small intestinal barrier function.

## Discussion

It is increasingly understood that the defensive properties of gel-forming mucins are important in protection against the development of human IBD (4), but the role of membrane mucins still remains elusive. In this study, we used a conditional mouse model to identify Muc17 as essential for the region-specific protection of the small intestine against commensal and pathogenic bacteria. Noticeably, a reduction in MUC17 levels in the enterocyte brush border and an impaired glycocalyx protection against bacteria were observed in the noninflamed ileum of patients with CD. Our findings suggest that membrane mucin and glycocalyx dysfunction precedes inflammation in CD.

The mechanisms underlying CD include genetic factors, such as *NOD2* mutations that impair the sensing and removal of intracellular bacteria (10). These defects in turn trigger immune responses that evoke chronic inflammation. Although there are no known disease-associated mutations in the *MUC17* gene and *MUC17* mRNA levels remain unaltered in the noninflamed ileum of patients with CD (31), studies have reported an altered glycocalyx ultrastructure in ileal CD (15). However, the implications of compromised MUC17 protein and glycocalyx barrier function in CD have not been addressed. In this study, we identified decreased MUC17 protein levels and a weakened glycocalyx in ileal CD to allow for increased bacteria-epithelium interactions. The results find mechanistic support in our earlier studies of murine small intestinal explants demonstrating that Muc17 forms the enterocyte glycocalyx (9). We also showed that the formation of the glycocalyx by Muc17 is induced by IL-22, a critical regulator of antibacterial defenses, including antimicrobial peptides (Reg3b and Reg3g) and mucin fucosylation via *FUT2* (32). Notably, many IBD risk genes, such as *IL23R*, *JAK1/2*, *TYK2*, and *STAT3*, participate in IL-22 signaling (32), and the frequency of IL-22–producing type 3 innate lymphoid cells is decreased in CD (33).

Investigations on the functional role of gastrointestinal membrane mucins in vivo remain sparse. MUC1/Muc1 is normally expressed in the human and murine stomach, and studies in *Muc1^–/–^* mice have shown a protective role in gastric *Helicobacter pylori* infection (34). MUC13 has been suggested to act as decoy for the SiiE adhesin of *Salmonella* *typhimurium*, and as a result *Muc13^–/–^* mice are susceptible to cecal infection by *Salmonella* *typhimurium* (35). Human MUC17 is confined to small intestinal enterocytes, while murine Muc17 is expressed in both the small and large intestines. We disentangled the segment-specific function using 2 interventions with distinct mechanisms of action. DSS disrupts the mucus layer separating gut bacteria from the epithelium (36). Accordingly, DSS-induced colitis is rapid and severe in mouse models with mucus defects (23–25). Since glycocalyx-deficient *Muc17^ΔIEC^* mice still produced a functional colonic mucus barrier, they were equally sensitive to colitis as wild-type controls. In an alternative intervention, we used *C*. *rodentium*, which shares virulence factors with enteropathogenic *Escherichia coli*, a major human pathogen that targets the small intestine. While *C*. *rodentium* is typically used to study pathogen-host interactions in the mouse distal colon (37), it surprisingly colonized the jejunum, including its mucosa, in Muc17-deficient mice. This finding highlights the crucial role of MUC17 in defending the small intestine against bacterial infections, even in the presence of a functioning mucus barrier. Interestingly, the ileum of Muc17-deficient mice was more resistant to colonization by *C*. *rodentium* than the jejunum. This difference can be explained by the lower expression of antimicrobial peptides in the mouse jejunum compared with the ileum, making it easier for the bacteria to colonize the jejunum (38). Given the established link between ileal CD and reduced antimicrobial peptide levels (39, 40), the murine jejunum emerges as a relevant model for ileal CD in humans.

Defects in intestinal defenses frequently result in the translocation of gut bacteria to peripheral tissues (26, 29). Bacterial translocation has also recently been linked to the expansion of “creeping fat” in CD (28). Under baseline conditions, *Muc17^ΔIEC^* mice exhibited an age-dependent spontaneous deterioration of epithelial homeostasis in the small intestine, involving elevated levels of host proteins participating in bacterial invasion (Arhgef26) and antimicrobial proteins such as Lyz1 and Reg3b. We attribute these host responses to increased and steadfast bacterial challenges in the absence of Muc17 and a functional glycocalyx. In line with these findings, *Muc17^ΔIEC^* mice displayed greater body mass, enlargement of abdominal fat, and viable commensal bacteria in peripheral tissues, all of which further support the importance of the glycocalyx-forming MUC17 in limiting pathology related to CD.

Gel-forming mucins not only serve as a physical barrier against bacteria but also provide a habitat for bacteria that utilize mucins as a carbon source (38, 41). However, such a role for membrane mucins has not been described. Our work shows that Muc17 regulates gut microbiota composition and signifies that Muc17-dependent changes in the bacterial community propagate from the small intestine, where Muc17 exerts its function, distally toward the colon. Importantly, increased abundance of Coriobacteria and Lachnoclostridium, enriched in *Muc17^ΔIEC^* mice, has also been reported in CD (42–44). Thus, our study suggests that MUC17 fulfills 2 purposes: forming a glycocalyx that blocks bacteria and regulating the selection of bacteria by the host. We postulate that the latter function depends on the apical localization of MUC17 on microvilli, which release luminal vesicles that bind and limit bacterial growth in the lumen (16). The absence of Muc17 from microvillus-derived luminal vesicles could alter vesicle interactions with bacteria, thereby impacting the microbial community.

In conclusion, we uncovered a role for the MUC17-based glycocalyx as an important defense system of the small intestine. Our findings shed light on the region-specific role of membrane mucins as innate defense components that create a critical interface between the host epithelium and the gut microbiota. The disruption of this system in the noninflamed ileum of CD suggests that defective MUC17 biosynthesis or trafficking is an early epithelial defect that precedes inflammation. Given the limited number of mouse models for small intestinal inflammation, the *Muc17^ΔIEC^* model provides opportunities for understanding the molecular mechanisms underlying epithelial cell dysfunction in CD.

## Methods

### Sex as a biological variable.

Our study examined female and male patients. We did not observe sex-specific differences. The study also examined male and female mice, and similar findings are reported for both sexes. Thus, sex was not considered as a biological variable.

### Experimental design.

We aimed to identify the molecular mechanisms by which compromised MUC17 and glycocalyx function impact disease development in ileal CD. To this end, noninflamed ileal biopsies from CD and non-IBD patients were analyzed for MUC17 levels and glycocalyx barrier integrity. Mechanistic and functional investigations were performed using a preclinical *Muc17^ΔIEC^* mouse strain, which was subjected to 2 distinct challenge models as well as analyzed longitudinally under baseline SPF conditions. Data collection and analysis were performed in a blinded manner whenever possible. For patient biopsies, sample size was not determined by power calculation but estimated based on our experience and previous studies. Sample sizes and statistical tests are described in the figure legends.

### Human participants.

Patients (≥18 years) with CD and individuals without suspected IBD (non-IBD), referred to Sahlgrenska University Hospital (Gothenburg, Sweden), were eligible for inclusion in the study. Clinical information and metadata for enrolled participants are provided in Supplemental Table 1. Patients with macroscopic or microscopic evidence of intestinal pathology other than CD were excluded. Biopsies were obtained during ileocolonoscopy, using biopsy forceps routinely used in standard of care. Three biopsies were obtained from the noninflamed terminal ileum of each participant and immediately placed into the appropriate vials: ice-cold oxygenated Krebs transport solution (45) for ex vivo glycocalyx permeability assay, 4% paraformaldehyde (PFA) for histology, and a dry vial frozen at –80°C.

### Mice.

The *Muc17^fl/fl^* mouse strain was engineered by introducing *loxP* sites flanking exons 3 and 5 of the *Muc17* gene (Ensembl gene ID: ENSMUSG00000037390; NCBI gene ID: 666339) (Supplemental Figure 2A). To generate the *Muc17^ΔIEC^* strain, *Muc17^fl/fl^* were crossed with *Vil1-Cre* mice (The Jackson Laboratory Strain 004586, RRID:IMSR_JAX:004586). All mice were on a C57BL/6N background, maintained under standardized SPF conditions of temperature (21°C–22°C) and illumination (12-hour light/12-hour dark cycle), with ad libitum access to food and water. Experimental groups consisted of age- and sex-matched 6- to 8-week-old cohoused littermates or 5-, 8-, or 36- to 40-week-old mice where indicated. Animals were anesthetized with isoflurane followed by cervical dislocation.

### Ex vivo glycocalyx permeability assay in ileal biopsies.

The barrier integrity of the glycocalyx was quantified as previously described (9). In detail, biopsies from the ileum of patients were collected in ice-cold oxygenated Krebs transport solution and pinned down with the epithelial side up in silicon-well plates. Mounted samples were stained with 50 μg/mL CellMask Deep Red plasma membrane stain (Thermo Fisher Scientific C10046) for 15 minutes at room temperature (RT), fixed with 4% PFA for 1 hour at RT, and washed 3 times with PBS. Bacterial penetration into the glycocalyx was quantified using viable *E*. *coli^GFP+^* (9), cultured overnight at 37°C in LB medium supplemented with 100 μg/mL ampicillin, and washed in PBS before the assay. Mounted biopsies were incubated with *E*. *coli^GFP+^* for 20 minutes prior to time-lapse confocal imaging (pixel dwell time 2.41 μsec, frame time 20.39 seconds) for 30 cycles using a Plan-Apochromat × 20/1.0 DIC water immersion objective (ZEISS), with 488/639 nm lasers, on an upright LSM 700 Axio Examiner Z.1 confocal imaging system (ZEISS) with Zen acquisition software (ZEISS). The spatial distance of individual *E*. *coli^GFP+^* cells relative to CellMask was extracted as a function of time using Imaris software (Oxford Instruments). The CellMask-stained brush border was reconstructed using the Isosurface function and shortest distance calculation. Individual *E*. *coli^GFP+^* cells were reconstructed using the Spot function and shortest distance calculation. The frequency distribution of *E*. *coli^GFP+^* cells in the distance interval of 0–2 μm from the CellMask-stained brush border was visualized in Prism 10 software (GraphPad). Quantification was performed on an average of 3 regions per villus and 3 villi per patient biopsy.

### Infection of mice with C. rodentium.

Streptomycin-resistant *C*. *rodentium* strain DBS100 (44) and chloramphenicol-resistant DBS100-derived *C*. *rodentium^GFP+^* (45) strains were used in the infection experiments. Infection inocula were cultured overnight in LB broth at 37°C in an orbital shaking incubator. Overnight cultures were concentrated 10-fold by centrifugation at 4,000 RCF for 10 minutes and resuspension in LB broth. Cohoused, female and male, 6- to 8-week-old *Muc17^fl/fl^* and *Muc17^ΔIEC^* littermates were gavaged with 200 μL of infection inoculum (2 × 10^8^ CFU). The endpoint of the experiment was as indicated in each experiment, loss of 10% of initial weight, or death. *C*. *rodentium* load at different tissue sites was determined by sacrificing the mice and collecting samples under aseptic conditions. Approximately 3 cm of jejunum, ileum, and DC were dissected and flushed with 4 mL sterile PBS to collect the contents. Contents and flushed tissue were collected separately. *C*. *rodentium* load was also quantified by sampling the liver, MLNs, and spleen. Tissue samples were homogenized in sterile PBS using an Ultra-Turrax T10 dispersing instrument (IKA) that was sequentially cleaned in 70% ethanol (×2) and sterile PBS. *C*. *rodentium* was enumerated from the homogenates by serial dilution on MacConkey agar plates supplemented with 100 μg/mL streptomycin or 30 μg/mL chloramphenicol, followed by overnight incubation at 37°C and quantification of bacterial CFU. For each sample, a theoretical LOD was calculated based on the detection of 1 colony at the lowest plated dilution. An average LOD is shown for each tissue sample.

### Statistics.

Statistical analysis and graphical illustrations were performed using Prism 10 software (GraphPad) and R open source software. Biological replicates for each group and experiment are stated in the figure legends. All data are presented as means ± SD except the quantification CFU of *C*. *rodentium* (Figure 4, D–F, and Supplemental Figure 5) as medians with interquartile range, quantification of total weight as median with minimum to maximum range of data points (Figure 5H), and quantification of the 16S rRNA gene in peripheral tissues (Figure 5J), which is represented as mean ± SEM. Statistical tests were applied as indicated in each figure legend, and the *P* values for each statistical test are indicated in each figure legend. *P* < 0.05 was considered statistically significant.

### Study approval.

Written informed consent was obtained from all patients in accordance with the respective protocol described in the ethical permit 2020-03196 approved by the Swedish Ethical Review Authority in Gothenburg, Sweden. The Swedish Laboratory Animal Ethical Committee in Gothenburg, Sweden (ethical permits 2285-19 and 2292-19), approved all experiments.

### Data availability.

All data are available in the main text or the supplemental material. Supporting Data Values for all the described experiments are reported in a supplemental Excel file. The mass spectrometry proteomics data have been deposited to the ProteomeXchange Consortium (http://proteomecentral.proteomexchange.org) via the PRIDE partner repository with the dataset identifier PXD048896.

Further information can be found in Supplemental Methods.

## Author contributions

EL and TP conceived the study. EL, SJ, LA, BD, and TP developed methodology. EL, SJ, FCVDK, MS, and TP performed investigation. EL and TP performed visualization. TP supervised. Materials were from SIMT, BAV, and TP. EL and TP wrote the original draft. EL, SJ, FCVDK, BD, LA, BAV, and TP reviewed and edited the manuscript.

## Supplementary Material

Supplemental data

Unedited blot and gel images

Supplemental table 1

Supplemental table 2

Supplemental table 3

Supplemental video 1

Supporting data values

## Version 1 12/19/2024

In-Press Preview

## Version 2 02/10/2025

Electronic publication
