# Supplementary material for: MUC17 is an essential small intestinal glycocalyx component that is disrupted in Crohn’s disease
Source: JCI Insight. 2024 Dec 19;10(3):e181481. doi: 10.1172/jci.insight.181481 (PMC11948581; doi:10.1172/jci.insight.181481)
Supplement: Supplemental data [file jciinsight-10-181481-s019.pdf]

Supplementary material for

MUC17 is an essential small intestinal glycocalyx component that is disrupted in Crohn's disease

Elena Layunta *et al.*

*Corresponding author. Email thaher.pelaseyed@medkem.gu.se

This PDF file includes:

Supplementary Methods

Supplemental Figures 1-10

Supplemental Table 1-4

Movie 1

Supplementary methods:

Genotyping using PCR

Ear punch biopsies or 3 mm tissue biopsies from mice were collected and frozen at -20°C. Genomic extraction was performed by addition of 65 µL of alkaline lysis buffer (25 mM NaOH, 0.2 mM EDTA, pH 12) and boiling at 95°C for 1 hour. Samples were neutralized with 65 µL 40 mM Tris-HCl, pH 5. 3 µL of the DNA extraction was used as a template in genotyping PCR reactions using HotStarTaq DNA polymerase (Qiagen Cat# 203205) and 10 µM of each primer. For genotyping of the two *loxP* sites in the *Muc17* gene, forward primer GH979F (5'-CAAACAGTGTCATACCCACTATGG-3') and reverse primer GH979R (5'-GGCTTTTGATGTTTGATTGTTG-3'), and forward primer GH980F (5'-CCTTAGAGGCATATTGTTCTCAGC-3') and reverse primer GH980R (5'-TGCCAGCATAAATCAGAGCC-3') were used (Supplemental Figure 2A). Thermocycling parameters were denaturation step at 95°C for 5 min., 35 cycles of 95°C for 30 s, 58°C for 50 s, 72°C for 3 min., and final elongation 72°C for 10 min.. For genotyping of the *Vil1-Cre* allele*,* forward primer 1878 (5'- GTGTGGGACAGAGAACAAACC-3') and reverse primer 1879 (5'- ACATCTTCAGGTTCTGCGGG-3') were used. Thermocycling parameters were denaturation step 95°C for 5 min., 35 cycles of 95°C for 30 s, 53°C for 50 s, 72°C for 1.5 min., and final elongation 72°C for 10 min..

Dextran sodium sulfate (DSS)-induced colitis

Six- to eight-week-old, cohoused female and male *Muc17*$^{fl/fl}$ and *Muc17*$^{\Delta IEC}$ littermates we subjected to *ad libitum* administration of 3% DSS (TdB Labs Cat# DB001) in the drinking water. The end point of the experiment was seven days of DSS treatment, loss of 10% of initial weight, or death. Stool consistency, hematochezia (fecal blood), and disease activity scores were calculated for animals that reached day six of treatment. The disease activity

index (DAI) was calculated as the sum of the combined scores for stool consistency, fecal blood, and weight loss (*43*). The detection of occult blood was performed using the Hemoccult Guaiac Fecal Occult Blood Test kit (Beckman Coulter. Cat# 61200A) according to the manufacturer's instructions. The probability of survival was defined by time of death or weight loss >10%. Jejunum and colon were dissected, and the colon length was measured from cecum to anus and normalized against the initial body weight of the respective animal. Segments of the jejunum and distal colon were fixed in Carnoy's fixative (60% absolute methanol, 30% chloroform, and 10% glacial acetic acid), prepared for sectioning, and stained for hematoxylin/eosin and Alcian Blue-PAS.

Calculation of relative risk

The relative risk of *C. rodentium* infection based on genotype and tissue was determined by calculating the ratio of the risk of *Muc17*$^{\Delta IEC}$ mice carrying *C. rodentium* counts above LOD to the risk of *Muc17*$^{fl/fl}$ mice carrying C. rodentium counts above LOD for each tissue site.

Tissue histology and immunohistochemistry

Human ileal biopsies or murine intestinal segments were fixed in Carnoy's fixative or 4% paraformaldehyde (PFA) solution. Fixed samples were embedded in paraffin, deparaffinized in xylene substitute (2 × 10 min., 60°C), and rehydrated in 100% ethanol (10 min.), 70% (v/v) ethanol (5 min.), 50% (v/v) ethanol (5 min.), and 30% (v/v) ethanol (5 min.). Sections were placed in antigen retrieval buffer (0.01M citric acid, pH 6.0) at 100 degrees for 10 min. and then cooled to RT (2 hours) and transferred to PBS. Tissues were enclosed with a hydrophobic PAP pen, permeabilized with 0.1% Triton X-100 in PBS for 5 min., and then blocked with 5% fetal calf serum (FCS) in PBS for 2 hours at room temperature (RT). Primary (overnight) and secondary antibodies (2 hours) were added to the dilution buffer (5%

FCS in PBS). Primary antibodies were rabbit anti-CDHR5 polyclonal antibody (1:250) (Atlas Antibodies Cat# HPA009081, RRID:AB_1079428), rabbit anti-Epcam polyclonal antibody (1:1000) (Abcam Cat# ab71916, RRID: AB_1603782), mouse anti-Ezrin monoclonal antibody (1:500) (Sigma-Aldrich Cat# E8897, RRID: AB_476955), mouse anti-GFP monoclonal antibody (1:500) (Sigma-Aldrich Cat# G6539, RRID: AB_259941), mouse anti-mKi67 monoclonal antibody (1:100) (Thermo Fisher Scientific Cat# 14-5698-82, RRID:AB_10854564), rabbit anti-Muc13 polyclonal antibody against human and mouse MUC13 (*46*) (1:500), rabbit anti-MUC17C1 polyclonal antibody against human MUC17 (*47*) (1:500), and rabbit anti-Muc17S2 polyclonal antibody against mouse Muc17 (*8*) (1:2,000). Secondary antibodies were donkey anti-rabbit IgG Alexa Fluor 488 (1:500) (Thermo Fisher Scientific Cat# A-21206 (also A21206), RRID: AB_2535792), goat anti-mouse IgG1 Alexa Fluor 555 (1:500) (Thermo Fisher Scientific Cat# A-21127, RRID: AB_2535769) and goat anti-rabbit Alexa Fluor 647 (1:500) (Thermo Fisher Scientific Cat# A-21245 (also A21245), RRID: AB_2535813). Brush border glycans were stained with 1 µg/mL biotinylated Aleuria aurantia lectin (AAL) (Vector Laboratories #B-1395-1) or wheat germ agglutinin (WGA) (Vector Laboratories #B-1025-5) overnight and incubated with Streptavidin Alexa Fluor 555 (1:500) (Thermo Fisher Scientific Cat# S21381, RRID:AB_2307336) for 2 hours. DNA was stained with 5 µg/mL Hoechst 34580 (ThermoFisher Scientific Cat# H21486) for 10 min.. Slides were washed three times with PBS after each incubation step. Coverslips were mounted using Prolong Gold antifade (ThermoFisher Scientific Cat# P36980) and polymerized for 2 hours at RT. Slides were imaged using an upright LSM 700 Axio Examiner Z.1 confocal imaging system (Carl Zeiss). AB-PAS stain was performed at pH 2.1-2.5. Assessment of tissue morphology, as measured by crypt number, villus and crypt length, and goblet cell number, in each group was performed in a blinded manner.

TUNEL assay

TUNEL assay was performed using In Situ Cell Death Detection Kit, TMR Red (Roche Cat# 12156792910). Rehydrated tissue sections on glass slides were subjected to antigen retrieval and permeabilization as described above. Following the blocking of sections with 3% BSA and 20% bovine serum in PBS for 30 min., slides were rinsed in PBS and sections were incubated with a TUNEL reaction mixture for 1 hour at 37°C in a humidified atmosphere in the dark. Negative control slides were prepared with label solution without terminal transferase. Positive control slides were pretreated with 3U /mL recombinant grade I DNase I in 50 mM Tris-HCl, pH 7.5, 10 mM $MgCl_2$, 1 mg/ml BSA for 10 min. at RT before incubation with TUNEL reaction mix. Slides were rinsed three times with PBS and stained with 5 µg/mL Hoechst 34580 for 10 min.. After three washes with PBS, slides were mounted as described above.

Bioorthogonal labeling of mucin *O*-glycans

Mice were intraperitoneally injected with 2.6 mg Tetraacetylated N-Azidoacetylgalactosamine (GalNAz) (ThermoFisher Scientific Cat# C33365) dissolved in 25 µL DMSO and diluted in a final volume of 500 µL PBS. Intestinal tissues were collected 8 hours after injection, fixed in 4% PFA, and prepared for paraffin sectioning. Sections were rehydrated as described previously. Click reaction was performed prior to blocking using the Click-IT™ Tetramethylrhodamine (TAMRA) Protein Analysis Detection Kit (ThermoFisher Scientific Cat# C33370) following the manufacturer's protocol. DNA was stained with 5 µg/mL Hoechst 34580 for 10 min. (ThermoFisher Scientific Cat# H21486).

***Ex vivo* quantification of mucus barrier properties in murine distal colon**

Quantification of mucus thickness and penetrability in the murine distal colon was performed as previously described (*48*). The distal colon was excised and flushed with ice-cold oxygenated Krebs buffer to remove luminal material, opened along the longitudinal axis, and mounted in a horizontal perfusion chamber (*49*). The tissue was overlaid with Krebs buffer containing a mixture of 5 µg/mL Hoechst 34580 (ThermoFisher Scientific Cat# H21486), 10 µg/mL UEAI Rhodamine (Vector Laboratories Cat# RL-1062-2), and 1 µm crimson carboxylate-modified FluoSpheres microbeads (1:20 dilution) (Thermo Fisher Scientific Cat# F8816) and incubated for 15 min.. The tissue was then washed with 0.5 mL Krebs buffer and submerged in 2 mL fresh Krebs buffer. Tissue incubated with microbeads was imaged using the 488/546/639-nm lasers on an LSM 700 Axio Examiner Z.1 confocal imaging system (Carl Zeiss) as described above. The colonic epithelium (Hoechst), mucus (UEAI), and microbead fluorescent signals were mapped using Imaris software (Oxford Instruments) and data describing the z-axis position of epithelium and microbeads was extracted. The thickness of the mucus layer was quantified by calculating the average epithelium-microbead z-axis distance. Normalized mucus penetrability was quantified by quantifying the distribution of microbeads within the mucus layer stained with UEAI. A frequency distribution curve of the axis distance of microbeads from the epithelium was generated for each z stack using Prism 10 software. Curves were normalized to maximum frequency values and then normalized to the position of the mucus surface and cropped to exclude data from microbeads above the mucus surface. Lastly, normalized penetrability was expressed as the area under the curve to allow for a quantitative comparison of microbead penetration between samples.

Isolation of luminal vesicles from intestinal segments

Isolation of luminal vesicles was adapted from (*50*). Briefly, mice were sacrificed as described above and the intestines were transferred to ice-cold saline (150 mM NaCl, 2 mM

imidazole-Cl) containing 0.02% NaN_3. The intestinal content was collected in a glass beaker by flushing the intestine with 30-60 mL of ice-cold saline. The content was centrifuged at 500 x g for 20 min., 20,000 x g for 30 min., and finally 100,000 x g for 2 hours. The supernatant from the proceeding centrifugation was used in the subsequent step. All centrifugation steps were conducted at 4°C. The final pellet, composed of luminal vesicles, was resuspended in PBS.

Electrophoresis and western blot

A suspension of luminal vesicles was reduced in 4X reducing sample buffer (8% SDS, 400 mM Dithiothreitol) and separated on precast 4%–12% SDS-polyacrylamide gel (ThermoFisher Scientific Cat# XP04125BOX). Proteins were transferred to a PVDF-FL membrane (Millipore Cat# IPFL00010) with a current of 2.5 mA/cm2 for 1 hour. The membrane was blocked in 5% non-fat milk in PBS for 30 min. and incubated with rabbit anti-Muc17C1 polyclonal antibody against mouse Muc17 (*51*) (1:250) in 5% non-fat milk in PBS + 0.1% Tween-20 (PBS-T) overnight at 4°C. The membrane was washed three times in PBS-T and incubated with Goat anti-rabbit Alexa Fluor 680 (1:20000) (Thermo Fisher Scientific Cat# A-21109, RRID:AB_2535758) secondary antibody for 1 hour at RT in the dark. The membrane was washed three times in PBS-T and visualized on an Odyssey CLx near-infrared fluorescence imaging system (LI-COR Biosciences).

16S rRNA gene sequencing for profiling of intestinal microbiota

DNA from luminal compartments was extracted by mechanical lysis using a Fast-Prep System with Lysing Matrix E tubes (MPBio) as previously described (*52*). Sample concentrations were measured using a Qubit instrument (Thermo Fisher Scientific Cat# Q33238) with the Qubit™ 1X dsDNA High Sensitivity (HS) (Thermo Fisher Scientific Cat#

Q33230). Luminal microbiota composition was profiled by sequencing the V3/V4 region of the 16S rRNA gene using a 2-step PCR approach (*53*). The forward and reverse primer mixes were prepared by combining eight phased primers each, at equimolar levels. For primer sequences see Supplemental Table 3. The PCR1 reaction was performed with 1 ng of input DNA, 10 µl KAPA HiFi Hotstart 2x Master Mix (Roche Cat# KK2602), 0.5 µl BSA (20 mg/ml), 1 µl of 7.5 µM forward primer mix and 1 µl of 7.5 µM reverse primer mix. Nuclease-free water was added to a reaction size of 21 µl. The PCR conditions for PCR1 were 98°C for 2 min., 20 cycles of 98°C for 20 s, 54°C for 20 s, 72°C for 15 s, and final elongation at 72°C for 2 min.. Samples were purified using 21 µl of MagSI NGSPrep plus purification beads (Magtivio Cat# MDKT0001) following the supplier's instructions and samples were eluted in 12 µl of Elution Buffer. 6 µl of the PCR1 eluate was subjected to amplification (PCR2 reaction) with the following reaction setup: 10 µl KAPA HiFi Hotstart 2x Master Mix, 1 µl of 5 µM Adapterama i7 index primer and 1 µl of 5 µM Adapterama i5 index primer. For primer sequences see Supplemental Table 3. Nuclease-free water was added to a reaction size of 20 µl. The PCR conditions for PCR2 were 98°C for 2 min., 8 cycles of 98°C for 20 s, 55°C for 30 s, 72°C for 30 s, and final elongation at 72°C for 2 min.. Samples were purified using 20 µl of MagSI NGSPrep plus purification beads. Libraries were pooled at equimolar levels and the molarity was determined by qPCR using the Illumina library Quantification kit from (Roche Cat# KK4824). Pools were run on a MiSeq V3 2×300 with a loading concentration of 11 pM. PhiX spike-in was 10%. The same library pool of phased libraries was used for all sequencing runs.

The 16S rRNA analysis was performed using the nf-core/ampliseq analysis pipeline (*54*). Bioinformatic analysis was performed with QIIME 2 2020.11 (*55*). Raw sequence data were demultiplexed and quality filtered followed by denoising with DADA2 (*56*). All amplicon sequence variants (ASVs) were aligned with mafft v.7.407 (*57*) and used to construct a

phylogeny with fastTree v.2.1.10 (*58*). Alpha-diversity metrics (Shannon diversity index), beta diversity metrics (Bray-Curtis dissimilarity), and Principal Coordinate Analysis (PCoA) were estimated using the diversity core-metrics-phylogenetic command. Taxonomy was assigned to ASVs using the q2-feature-classifier (*59*) classify-Sklearn naïve Bayes taxonomy classifier against the Silva v.138 reference sequence database (*60*). LEfSe algorithm was used to correlate genus-level relative abundance data to different experimental groups (*61*). Microbial features in the luminal compartment of each genotype were correlated to the calculated z-scores representing the relative abundance of each taxon along the gastrointestinal tracts reported by Lkhagva et al. (*27*).

Quantification of bacteria by 16S rRNA gene qPCR

For assessment of bacterial density in peripheral tissues, tissues were dissected and flushed with 5 mL 0.22 µm filter-sterilized PBS. Tissues were lysed by brief homogenization using an Ultra-Turrax T10 dispersing instrument (IKA) that was cleaned in RBS detergent (Merck), 2 times in 70% ethanol, and filter-sterilized ddH2O between each sample. Tissue lysates were centrifuged at 10,000 RCF for 10 min. to pellet bacterial cells and tissue debris. gDNA was isolated from small intestine mesenteric lymph nodes (MLNs), distal colon MLNs, spleen, and liver of mice using QIAamp PowerFecal Pro DNA kit (Qiagen Cat# 51804) and the accompanying protocol. Sample homogenization was performed on FastPrep-24 (MP Biomedicals). DNA extractions were quantified using a Nanodrop spectrophotometer (Thermo Fischer Scientific). Peripheral tissues were expected to have low 16S copy numbers, thus a limited cycle number (LCN) PCR was performed to amplify the whole 16S gene. 500 ng extracted gDNA was amplified using Platinum Green Hot Start PCR Master Mix (Thermo Fischer Scientific Cat# 13001012), 0.2µM universal whole 16S gene forward and reverse primers 27F (5’-AGAGTTTGATYMTGGCTCAG-3’) and 1492R (5’-

GGTTACCTTGTTACGACTT-3’). For samples with low concentrations of gDNA, 10µL was added to the LCN PCR reactions. 16S rRNA gene standards prepared from E. coli, contamination controls (PBS used to isolate the gDNA), and a blank were also amplified. Thermocycling conditions were initial denaturation step at 95°C for 5 min, 16 cycles of 94°C for 1 min, 55°C for 1 min, 72°C for 1.5 min, and 72°C for 10 min. After the LCN PCRs, the samples, standards, and contamination controls were analyzed by qPCR for 16S quantification using SsoAdvanced Universal SYBR Green Supermix (BioRad Cat# 1725270), 0.5µM universal forward and reverse primers 926F (5’-AAACTCAAAKGAATTGACGG-3’) and 1062R (5’-CTCACRRCACGAGCTGAC-3’), and 2µL LCN PCR product per reaction. qPCRs were run on the CFX96 qPCR (Bio-Rad), with the following protocol: 95°C for 5 min, 49 cycles of 95°C for 15 s, 61.5°C for 15 s, 72°C for 20 s, and 72°C for 5 min, finishing with a melt curve step of 65°C to 95°C in 0.5°C increments for 5 s. Data was analyzed in Bio-Rad CFX Manager software, whereby the standards were used as a calibration curve for the quantification of the samples and contamination controls. Data was quantified to copies/mL and normalized to weight of the tissue.

RNA extraction and qRT-PCR

Tissue from mouse jejunum tissue was persevered in RNAprotect Tissue Reagent (Qiagen Cat# 76104) and lysed in RLT buffer using an Ultra-Turrax rotor-stator homogenizer (IKA Werke). Tissue RNA was extracted using RNeasy Mini columns (Qiagen Cat# 74104) according to the manufacturer’s instructions and eluted into RNase-free H2O. The expression of *Il-1b*, *Il-6*, *Nfkb2*, and *Tnfa* genes was analyzed by qRT-PCR of cDNA prepared from 2 µg extracted RNA using the High-Capacity cDNA Reverse Transcription Kit (ThermoFisher Cat# 4368814). PCRs (20 µl) were prepared using SsoAdvanced Universal SYBR Green

Supermix (Bio-Rad Cat# 1725274), 450 nM forward and 450 nM reverse primers, and 25 ng cDNA. PCR cycling conditions were 95°C for 30 s and 40× cycles of 95°C for 15 s and 60°C for 30 s. Gene expression was quantified using pre-validated primers (TaqMan™ Array Mouse Immune Response, ThermoFisher Cat# 4414079) for *Il-1b* (Mm00434228_m1), *Il-6* (Mm00446190_m1), *Nfkb2* (Mm00479807_m1), and *Tnfa* (Mm00443258_m1). Reactions were monitored using a CFX96 platform (Bio-Rad) and calculations performed using the ΔΔCq method with data normalized to the housekeeping genes *18S* (Hs99999901_s1), *Gapdh* (Mm99999915_g1), *Hprt1* (Mm00446968_m1), and *Gusb* (Mm00446953_m1).

Fluorescence *in situ* hybridization

FISH staining for the bacterial 16S rRNA was performed on tissue sections from Si5 and DC. Sections were deparaffinized, air dried, and incubated with hybridization buffer (40% vol/vol formamide, 0.1% wt/vol SDS, 0.9 M NaCl, and 20 mM Tris, pH 7.4) supplemented with 1 mM Alexa 555-labeled universal bacterial FISH probe EUB338 (*62*). Slides were incubated at 37°C overnight in a RapidFISH Slide Hybridization Oven (Boekel Scientific), rinsed in wash buffer (0.9 M NaCl and 25 mM Tris, pH 7.4), and incubated for 20 min. at 50°C. Finally, slides were rinsed in double-distilled water and counterstained with 5 μg/mL Hoechst DNA stain and Wheat Germ Agglutinin Fluorescein (WGA) (10 μg/mL) (Vector Laboratories Cat# FL-1021) for 15 min. before the mounting of coverslips. Slides were imaged with an LSM700 confocal microscope (Zeiss).

Plate culture and identification of viable bacteria in extraintestinal tissues

Liver, mesenteric lymph nodes, and spleen were harvested into 1 mL sterile PBS and homogenized by a sterilized 5 mm stainless steel bead for 40 sec using a Fast-Prep 24 instrument (MP Biomedicals). 100 μL homogenate of each tissue was spread on brain heart

infusion supplemented (BHIS) agar plates and incubated at 37˚C under anaerobic conditions for 5 days. Colonies were transferred to 100 µL ddH2O and the bacterial 16S rRNA gene was amplified with the primer 27F (5'-AGAGTTTGATYMTGGCTCAG-3') and 1492R (5'-GGTTACCTTGTTACGACTT-3') using *PfuUltra* High-Fidelity DNA Polymerase (Agilent Cat# #600380). Thermocycling conditions were initial denaturation step of 95°C for 1 min., 35 cycles of 95°C for 30 s, 48°C for 30 s and 72°C for 2 min., and an extension step 72°C for 10 min.. Amplicons were separated on 1.5% agarose gel, excised and purified using a Nucleospin PCR Clean Up kit (Macherey Nagel Cat# 740609.50) and subjected to DNA sequencing (Eurofins Genomics) using sequencing primers targeting the hypervariable region V3-V4 of the 16S rRNA gene, S-D-Bact-0341-b-S-17(5'-CCTACGGGNGGCWGCAG-3') and the S-D-Bact-0785-a-A-21 (5'-GACTACHVGGGTATCTAATCC-3') (*63*). 16S gene sequences were analyzed by the BLASTn tool (NCBI) to identify bacterial isolates.

Single-cell analysis of published data sets

Single-cell sequencing data of the human small intestine and colon were extracted from GSE185224 (*64*) using the Gene Expression tool to query CZ CELLxGENE Discover (Chan Zuckerberg Initiative). Single-cell sequencing data of the mouse small intestine was obtained from GSE92332 (*65*) and extracted from the Single Cell Portal (Broad Institute).

Proteomic profiling of intestinal epithelial cells

Jejunal segments were opened longitudinally, washed in PBS for 5 min., and then incubated in PBS containing 3 mm EDTA and 1 mm DTT at 4 °C for 1 hour while gently shaken. The solution was replaced with fresh PBS, and epithelial cells were dissociated from the tissue by vigorous shaking for 30 s. The remaining tissue was removed from the solution using forceps, and cells were pelleted via centrifugation at 500 rpm. Isolated epithelial cells were

lysed in 400 µL lysis buffer (100 mM DTT, 4% SDS, 100 mM Tris pH 7.5) and heated at 95°C for 5 min.. Lysates were sonicated for 10 s, centrifuged at 16,000 x g at RT and the supernatant was added onto 10 kDa cutoff filters (PALL Cat# OD010C33). Proteins were digested using filter-aided sample preparation (*66*) with trypsin at 37°C overnight. Peptide concentration after elution was measured at 280 nm using NanoDrop (Thermo Fisher Scientific) and peptides were cleaned with StageTip C18 columns (*67*) before mass spectrometry (MS).

Nano LC-MS/MS was performed on a Q-Exactive HF mass-spectrometer (Thermo Fischer Scientific), connected with an EASY-nLC 1000 system (Thermo Fischer Scientific) through a nanoelectrospray ion source. Peptides were loaded on a reverse-phase column (150 mm x 0.075 mm inner diameter, New Objective, New Objective, Woburn, MA), packed in-house with Reprosil-Pur C18-AQ 3 mm particles (Dr. Maisch, Ammerbuch, Germany). Peptides were separated with a 230-minute gradient: from 3% to 25% B in 175 min., 25% to 45% B in 30 min., 45% to 100% B in 5 min., followed 20 min. wash with 100% of B (A: 0.1% formic acid, B: 0.1% formic acid/80% acetonitrile) using a flow rate of 250 nl/min.. Q-Exactive HF was operated at 250°C capillary temperature and 2.0 kV spray voltage. Full mass spectra were acquired in the Orbitrap mass analyzer over a mass range from m/z 350 to 1600 with a resolution of 60 000 (m/z 200) after the accumulation of ions to a 3e6 target value based on predictive AGC from the previous full scan. The twelve most intense peaks with a charge state ≥ 2 were fragmented in the HCD collision cell with a normalized collision energy of 27%. The tandem mass spectrum was acquired in the Orbitrap mass analyzer with the resolution of 15,000 after accumulation of ions to a 1e5 target value. Dynamic exclusion was set to 30 s. The maximum allowed ion accumulation times were 20 ms for full MS scans and 50 ms for tandem mass spectrum.

MS raw files were analyzed with MaxQuant software version 1.5.7.4 (*68*). Peak lists were identified by searching against the mouse UniProt protein database (downloaded 2022.11.11), supplemented with an in-house database containing all the mouse mucin sequences (http://www.medkem.gu.se/mucinbiology/databases/). Searches were performed using trypsin as an enzyme, maximum 2 missed cleavages, and a precursor tolerance of 20 ppm in the first search used for recalibration, followed by 7 ppm for the main search and 0.5 Da for fragment ions. Carbamidomethylation of cysteine was set as a fixed modification, and methionine oxidation and protein N-terminal acetylation were set as variable modifications. The required false discovery rate (FDR) was set to 1% for peptide and protein levels, and the minimum required peptide length was set to seven amino acids. Label-free quantification (LFQ) was based on two peptides. Raw mass spectrometry data were analyzed with DEP package (*69*) for R. Briefly, intensities were background corrected and normalized by variance stabilizing transformation and filtered based on valid values criteria (a minimum of 3 valid values in at least one of the groups). Missing values were imputed with a width of 0.3 of the Gaussian distribution relative to the standard deviation of measured values and a downshift of 1.8 units of the standard deviation of the valid data. Volcano plots were generated to visualize differentially expressed proteins between two conditions.

Supplementary references

42. H. A. H. Krebs, K., Untersuchungen uber die Harnstoffbildung im Tierkörper. *Biological Chemistry* **210**, 33-66 (1932).

43. D. J. Friedman, B. M. Kunzli, A. R. YI, J. Sevigny, P. O. Berberat, K. Enjyoji, E. Csizmadia, H. Friess, S. C. Robson, From the Cover: CD39 deletion exacerbates experimental murine colitis and human polymorphisms increase susceptibility to inflammatory bowel disease. *Proc Natl Acad Sci U S A* **106**, 16788-16793 (2009).

44. D. B. Schauer, S. Falkow, Attaching and effacing locus of a Citrobacter freundii biotype that causes transmissible murine colonic hyperplasia. *Infect Immun* **61**, 2486-2492 (1993).

45. K. S. Bergstrom, V. Kissoon-Singh, D. L. Gibson, C. Ma, M. Montero, H. P. Sham, N. Ryz, T. Huang, A. Velcich, B. B. Finlay, K. Chadee, B. A. Vallance, Muc2 protects against lethal infectious colitis by disassociating pathogenic and commensal bacteria from the colonic mucosa. *PLoS Pathog* **6**, e1000902 (2010).

46. M. D. Walsh, J. P. Young, B. A. Leggett, S. H. Williams, J. R. Jass, M. A. McGuckin, The MUC13 cell surface mucin is highly expressed by human colorectal carcinomas. *Hum Pathol* **38**, 883-892 (2007).

47. H. Schneider, E. Berger, B. Dolan, B. Martinez-Abad, L. Arike, T. Pelaseyed, G. C. Hansson, The human transmembrane mucin MUC17 responds to TNFalpha by increased presentation at the plasma membrane. *Biochem J* **476**, 2281-2295 (2019).

48. J. K. Volk, E. E. L. Nystrom, S. van der Post, B. M. Abad, B. O. Schroeder, A. Johansson, F. Svensson, S. Javerfelt, M. E. V. Johansson, G. C. Hansson, G. M. H. Birchenough, The Nlrp6 inflammasome is not required for baseline colonic inner mucus layer formation or function. *J Exp Med* **216**, 2602-2618 (2019).

49. J. K. Gustafsson, A. Ermund, M. E. V. Johansson, A. Schütte, G. C. Hansson, H. Sjövall, An ex vivo method for studying mucus formation, properties, and thickness in human colonic biopsies and mouse small and large intestinal explants. *Am J Physiol-Gastrointest Liver Physiol* **302**, G430-G438 (2012).

50. R. E. McConnell, J. N. Higginbotham, D. A. Shifrin Jr, D. L. Tabb, R. J. Coffey, M. J. Tyska, The enterocyte microvillus is a vesicle-generating organelle. *J Cell Biol* **185**, 1285-1298 (2009).

51. E. K. Malmberg, K. A. Noaksson, M. Phillipson, M. E. Johansson, M. Hinojosa-Kurtzberg, L. Holm, S. J. Gendler, G. C. Hansson, Increased levels of mucins in the cystic fibrosis mouse small intestine, and modulator effects of the Muc1 mucin expression. *Am J Physiol Gastrointest Liver Physiol* **291**, G203-210 (2006).

52. A. Salonen, J. Nikkila, J. Jalanka-Tuovinen, O. Immonen, M. Rajilic-Stojanovic, R. A. Kekkonen, A. Palva, W. M. de Vos, Comparative analysis of fecal DNA extraction methods with phylogenetic microarray: effective recovery of bacterial and archaeal DNA using mechanical cell lysis. *J Microbiol Methods* **81**, 127-134 (2010).

53. L. W. Hugerth, H. A. Wefer, S. Lundin, H. E. Jakobsson, M. Lindberg, S. Rodin, L. Engstrand, A. F. Andersson, DegePrime, a program for degenerate primer design for broad-taxonomic-range PCR in microbial ecology studies. *Appl Environ Microbiol* **80**, 5116-5123 (2014).

54. D. Straub, N. Blackwell, A. Langarica-Fuentes, A. Peltzer, S. Nahnsen, S. Kleindienst, Interpretations of Environmental Microbial Community Studies Are Biased by the Selected 16S rRNA (Gene) Amplicon Sequencing Pipeline. *Front Microbiol* **11**, 550420 (2020).

55. E. Bolyen, J. R. Rideout, M. R. Dillon, N. A. Bokulich, C. C. Abnet, G. A. Al-Ghalith, H. Alexander, E. J. Alm, M. Arumugam, F. Asnicar, Y. Bai, J. E. Bisanz, K. Bittinger, A. Brejnrod, C. J. Brislawn, C. T. Brown, B. J. Callahan, A. M. Caraballo-Rodriguez, J. Chase, E. K. Cope, R. Da Silva, C. Diener, P. C. Dorrestein, G. M. Douglas, D. M. Durall, C. Duvallet, C. F. Edwardson, M. Ernst, M. Estaki, J. Fouquier, J. M. Gauglitz, S. M. Gibbons, D. L. Gibson, A. Gonzalez, K. Gorlick, J. Guo, B. Hillmann, S. Holmes, H. Holste, C. Huttenhower, G. A. Huttley, S. Janssen, A. K. Jarmusch, L. Jiang, B. D. Kaehler, K. B. Kang, C. R. Keefe, P. Keim, S. T. Kelley, D. Knights, I. Koester, T. Kosciolek, J. Kreps, M. G. I. Langille, J. Lee, R. Ley, Y. X. Liu, E.

Loftfield, C. Lozupone, M. Maher, C. Marotz, B. D. Martin, D. McDonald, L. J. McIver, A. V. Melnik, J. L. Metcalf, S. C. Morgan, J. T. Morton, A. T. Naimey, J. A. Navas-Molina, L. F. Nothias, S. B. Orchanian, T. Pearson, S. L. Peoples, D. Petras, M. L. Preuss, E. Pruesse, L. B. Rasmussen, A. Rivers, M. S. Robeson, 2nd, P. Rosenthal, N. Segata, M. Shaffer, A. Shiffer, R. Sinha, S. J. Song, J. R. Spear, A. D. Swafford, L. R. Thompson, P. J. Torres, P. Trinh, A. Tripathi, P. J. Turnbaugh, S. Ul-Hasan, J. J. J. van der Hooft, F. Vargas, Y. Vazquez-Baeza, E. Vogtmann, M. von Hippel, W. Walters, Y. Wan, M. Wang, J. Warren, K. C. Weber, C. H. D. Williamson, A. D. Willis, Z. Z. Xu, J. R. Zaneveld, Y. Zhang, Q. Zhu, R. Knight, J. G. Caporaso, Reproducible, interactive, scalable and extensible microbiome data science using QIIME 2. *Nat Biotechnol* **37**, 852-857 (2019).

56. B. J. Callahan, P. J. McMurdie, M. J. Rosen, A. W. Han, A. J. Johnson, S. P. Holmes, DADA2: High-resolution sample inference from Illumina amplicon data. *Nat Methods* **13**, 581-583 (2016).

57. K. Katoh, K. Misawa, K. Kuma, T. Miyata, MAFFT: a novel method for rapid multiple sequence alignment based on fast Fourier transform. *Nucleic Acids Res* **30**, 3059-3066 (2002).

58. M. N. Price, P. S. Dehal, A. P. Arkin, FastTree 2--approximately maximum-likelihood trees for large alignments. *PLoS One* **5**, e9490 (2010).

59. N. A. Bokulich, B. D. Kaehler, J. R. Rideout, M. Dillon, E. Bolyen, R. Knight, G. A. Huttley, J. Gregory Caporaso, Optimizing taxonomic classification of marker-gene amplicon sequences with QIIME 2's q2-feature-classifier plugin. *Microbiome* **6**, 90 (2018).

60. C. Quast, E. Pruesse, P. Yilmaz, J. Gerken, T. Schweer, P. Yarza, J. Peplies, F. O. Glockner, The SILVA ribosomal RNA gene database project: improved data processing and web-based tools. *Nucleic Acids Res* **41**, D590-596 (2013).

61. N. Segata, J. Izard, L. Waldron, D. Gevers, L. Miropolsky, W. S. Garrett, C. Huttenhower, Metagenomic biomarker discovery and explanation. *Genome Biol* **12**, R60 (2011).

62. R. I. Amann, B. J. Binder, R. J. Olson, S. W. Chisholm, R. Devereux, D. A. Stahl, Combination of 16S rRNA-targeted oligonucleotide probes with flow cytometry for analyzing mixed microbial populations. *Appl Environ Microbiol* **56**, 1919-1925 (1990).

63. A. Klindworth, E. Pruesse, T. Schweer, J. Peplies, C. Quast, M. Horn, F. O. Glockner, Evaluation of general 16S ribosomal RNA gene PCR primers for classical and next-generation sequencing-based diversity studies. *Nucleic Acids Res* **41**, e1 (2013).

64. J. Burclaff, R. J. Bliton, K. A. Breau, M. T. Ok, I. Gomez-Martinez, J. S. Ranek, A. P. Bhatt, J. E. Purvis, J. T. Woosley, S. T. Magness, A Proximal-to-Distal Survey of Healthy Adult Human Small Intestine and Colon Epithelium by Single-Cell Transcriptomics. *Cell Mol Gastroenterol Hepatol* **13**, 1554-1589 (2022).

65. A. L. Haber, M. Biton, N. Rogel, R. H. Herbst, K. Shekhar, C. Smillie, G. Burgin, T. M. Delorey, M. R. Howitt, Y. Katz, I. Tirosh, S. Beyaz, D. Dionne, M. Zhang, R. Raychowdhury, W. S. Garrett, O. Rozenblatt-Rosen, H. N. Shi, O. Yilmaz, R. J. Xavier, A. Regev, A single-cell survey of the small intestinal epithelium. *Nature* **551**, 333-339 (2017).

66. J. R. Wisniewski, A. Zougman, N. Nagaraj, M. Mann, Universal sample preparation method for proteome analysis. *Nat Methods* **6**, 359-362 (2009).

67. J. Rappsilber, M. Mann, Y. Ishihama, Protocol for micro-purification, enrichment, pre-fractionation and storage of peptides for proteomics using StageTips. *Nat Protoc* **2**, 1896-1906 (2007).

68. J. Cox, M. Mann, MaxQuant enables high peptide identification rates, individualized p.p.b.-range mass accuracies and proteome-wide protein quantification. *Nat Biotechnol* **26**, 1367-1372 (2008).

69. X. Zhang, A. H. Smits, G. B. van Tilburg, H. Ovaa, W. Huber, M. Vermeulen, Proteome-wide identification of ubiquitin interactions using UbIA-MS. *Nat Protoc* **13**, 530-550 (2018).

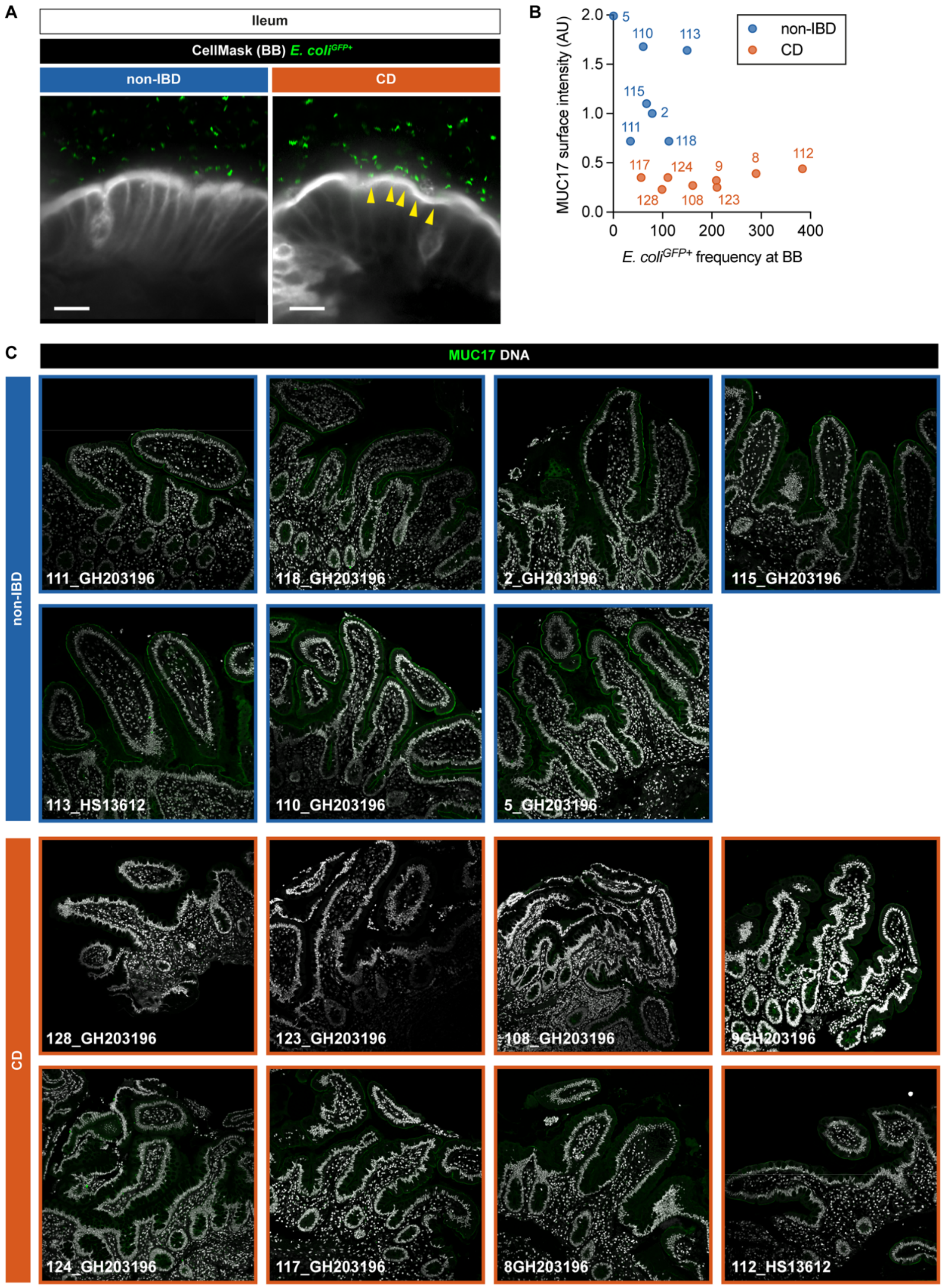

Supplemental Figure 1. Confocal micrographs of biopsies from non-IBD and CD patients analyzed using *ex vivo* glycocalyx permeability assay.

(A) Representative confocal micrographs of biopsy explants, stained for the brush border (CellMask, white) and incubated with *E. coli*$^{GFP+}$ (green). Yellow arrows indicate points of contact between *E. coli*$^{GFP+}$ and the brush border. Scale bar 10 μm. **(B)** Correlation analysis of MUC17 surface intensity and *E. coli*$^{GFP+}$ frequency at the brush border (BB) in ileal biopsies of non-IBD and CD patients, presented in Figure 1F. **(C)** Confocal micrographs of biopsies analyzed with *ex vivo* glycocalyx permeability assay in Figure 1F and analyzed in Supplemental Figure 1B, stained for MUC17 (green) and DNA (gray).

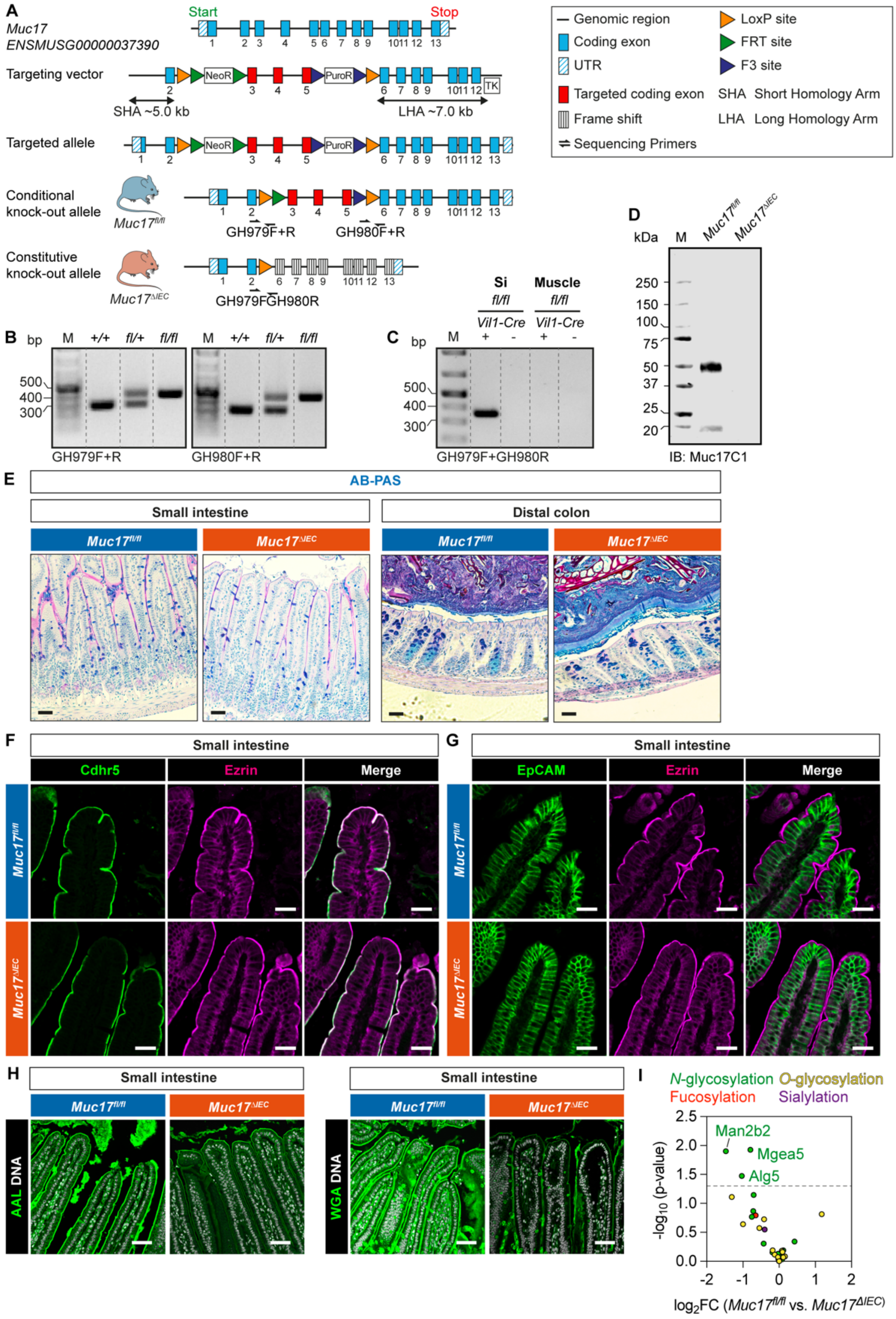

Supplemental Figure 2. Characterization of the *Muc17*$^{\Delta IEC}$ mouse strain.

(A) Schematic illustration of the murine *Muc17* gene and the strategy for introducing *loxP* sites after the exons 2 and 5. The binding sites for primers GH979F+R and GH980F+R are indicated with black arrows. **(B)** Representative DNA agarose gels showing genotyping of the floxed *Muc17* allele. M, molecular weight. **(C)** Representative DNA agarose gels showing genotyping of the floxed *Muc17* alleles in the presence and absence of the *Vil1-Cre* allele in the small intestine (Si) and muscle tissues. M, molecular weight. **(D)** Immunoblot (IB) of luminal vesicles isolated from the small intestine of $Muc17^{fl/fl}$ and $Muc17^{\Delta IEC}$ mice using the MU17C1 antibody. **(E)** AB-PAS staining of the jejunum (Si5) and distal colon (DC) of $Muc17^{fl/fl}$ and $Muc17^{\Delta IEC}$ mice. Scale bar 50 µm. **(F)** Immunohistochemistry of Cdhr5 (green) and Ezrin (magenta) in histological sections of the small intestine in $Muc17^{fl/fl}$ and $Muc17^{\Delta IEC}$ mice. Scale bar 20 µm. (**G**) Immunohistochemistry of Epcam (green) and Ezrin (magenta) in histological sections of the small intestine in $Muc17^{fl/fl}$ and $Muc17^{\Delta IEC}$ mice. Scale bar 20 µm. (**H**) Immunohistochemistry using the lectins AAL and WGA in the small intestine of $Muc17^{fl/fl}$ and $Muc17^{\Delta IEC}$ mice. Scale bar 50 µm. (**I**) Volcano plots showing the fold change of protein expression of detected glycosyltransferases and associated proteins in epithelial cells of the jejunum (Si5) from 5-week-old $Muc17^{fl/fl}$ compared to $Muc17^{\Delta IEC}$ mice. No differentially expressed proteins (fold change ≥ 2, p-value < 0.05) were detected. Specific proteins are labeled with their protein names. n=5 for each genotype.

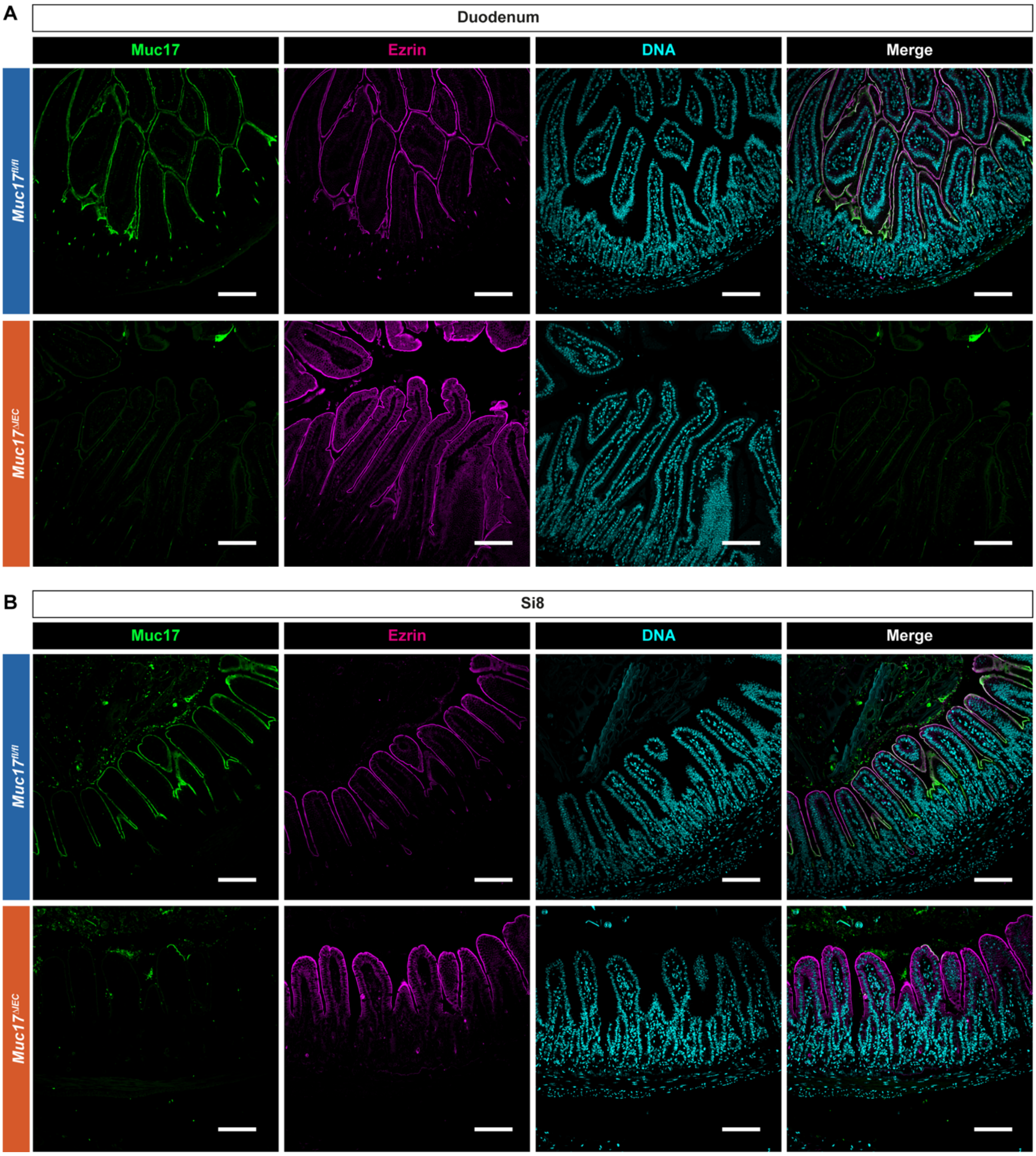

Supplemental Figure 3. Confocal micrographs of the small intestine of *Muc17*$^{fl/fl}$ and *Muc17*$^{\Delta IEC}$ mice.

(A) Immunohistochemistry of Muc17 (green), Ezrin (magenta) and DNA (cyan) in histological sections from the duodenum of *Muc17*$^{fl/fl}$ and *Muc17*$^{\Delta IEC}$ mice. Scale bar 100 µm.

(B) Immunohistochemistry of Muc17 (green), Ezrin (magenta) and DNA (cyan) in histological sections from the ileum (Si8) of *Muc17*$^{fl/fl}$ and *Muc17*$^{\Delta IEC}$ mice. Scale bar 100 µm.

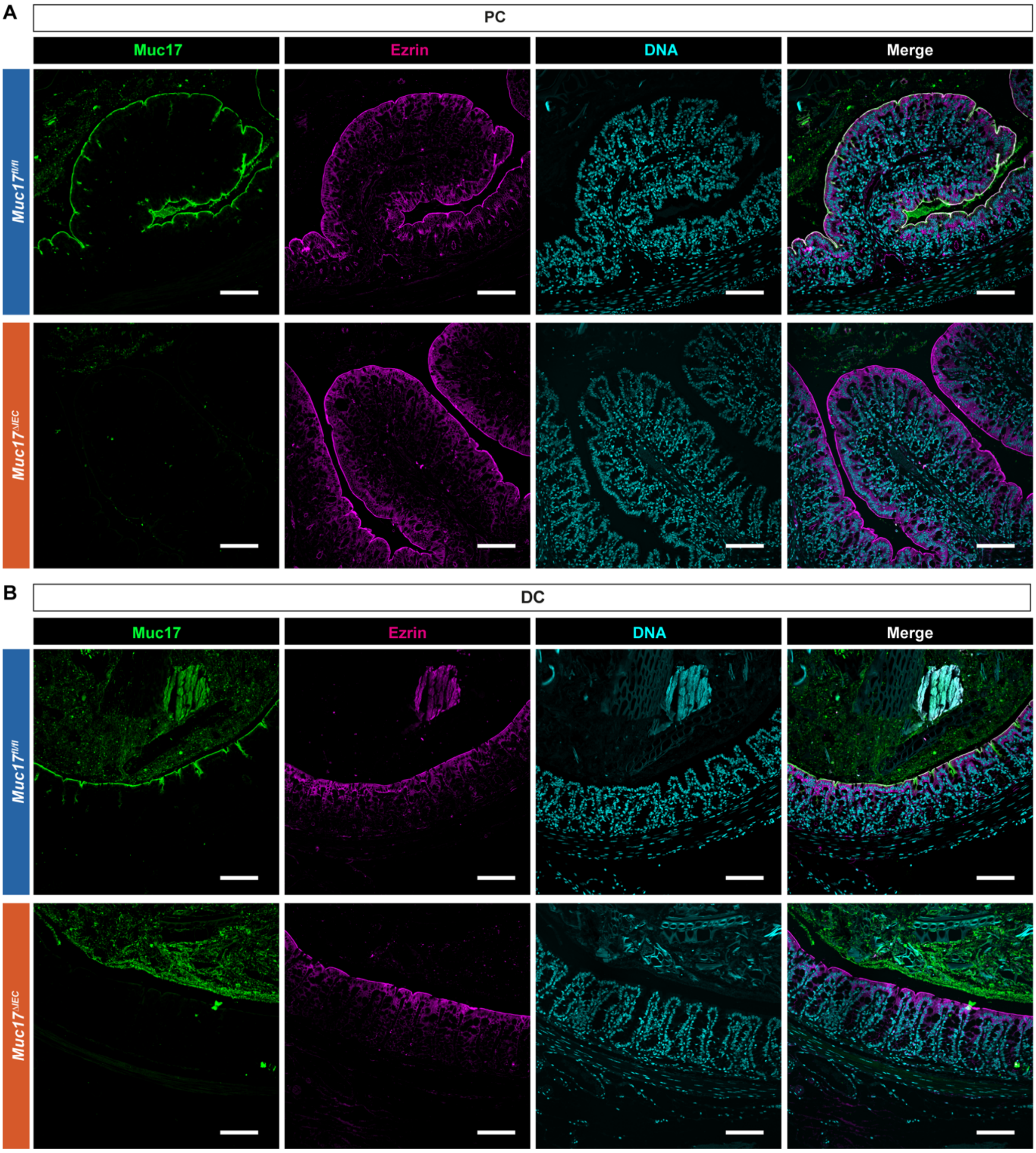

Supplemental Figure 4. Confocal micrograph of the colon of *Muc17*$^{fl/fl}$ and *Muc17*$^{\Delta IEC}$ mice.

(A) Immunohistochemistry of Muc17 (green), Ezrin (magenta) and DNA (cyan) in histological sections from the proximal colon (PC) of *Muc17*$^{fl/fl}$ and *Muc17*$^{\Delta IEC}$ mice. Scale bar 100 µm. **(B)** Immunohistochemistry of Muc17 (green), Ezrin (magenta) and DNA (cyan) in histological sections from the distal colon (DC) of *Muc17*$^{fl/fl}$ and *Muc17*$^{\Delta IEC}$ mice. Scale bar 100 µm.

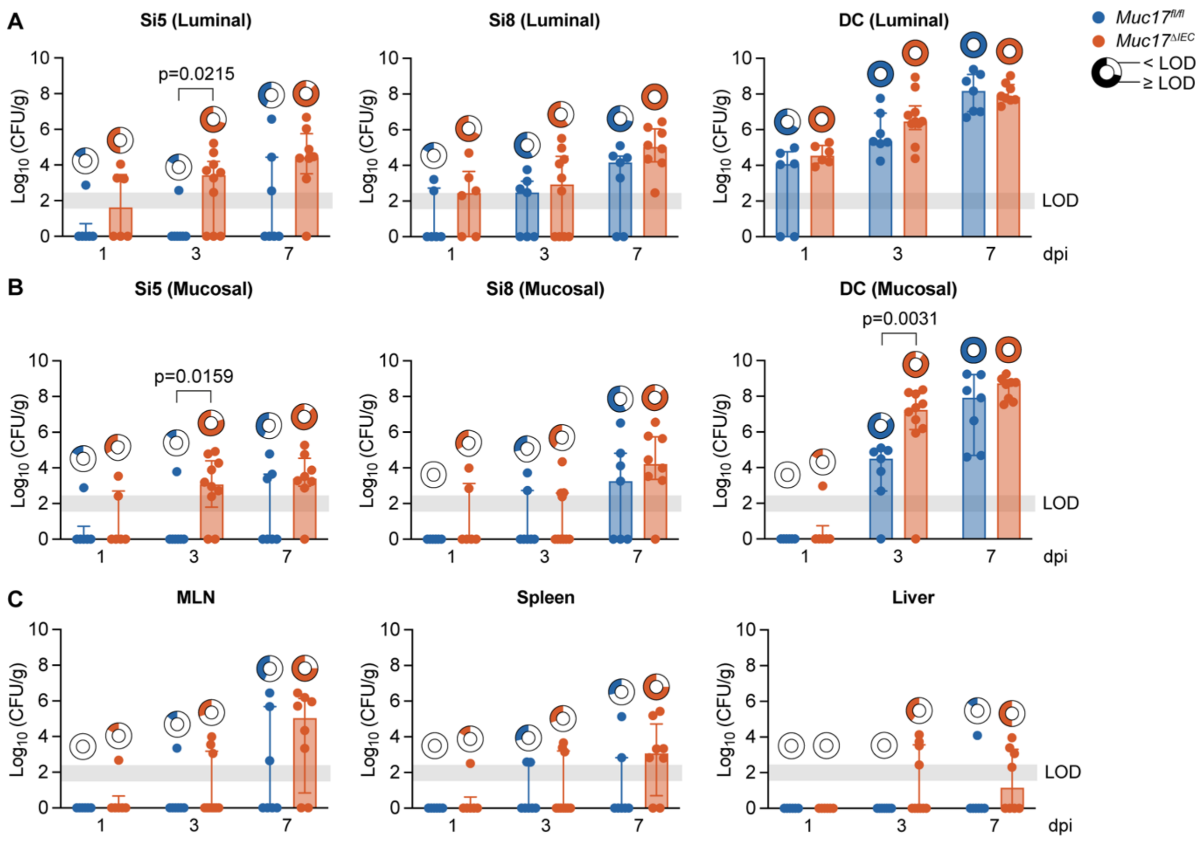

Supplemental Figure 5. Longitudinal analysis of *C. rodentium* infection in *Muc17$^{fl/fl}$* and *Muc17$^{\Delta IEC}$* mice.

(A) Quantification of *C. rodentium* colony forming unites (CFU) in the luminal compartment of the jejunum (Si5), ileum (Si8), and distal colon (DC) of *Muc17$^{fl/fl}$* and *Muc17$^{\Delta IEC}$* mice at 1, 3, and 7 days post-infection (dpi). **(B)** Quantification of *C. rodentium* CFU in the mucosal compartment of Si5, Si8, and DC of *Muc17$^{fl/fl}$* and *Muc17$^{\Delta IEC}$* mice at 1, 3, and 7 dpi. (**C**) Quantification of *C. rodentium* CFU in the mesenteric lymph nodes (MLN), spleen, and liver of *Muc17$^{fl/fl}$* and *Muc17$^{\Delta IEC}$* mice at 1, 3, and 7 dpi. The circle charts represent the proportion of mice carrying *C. rodentium* CFU above the limit of detection (LOD) in each segment of each genotype. Significance was determined by Mann-Whitney test (A-B).

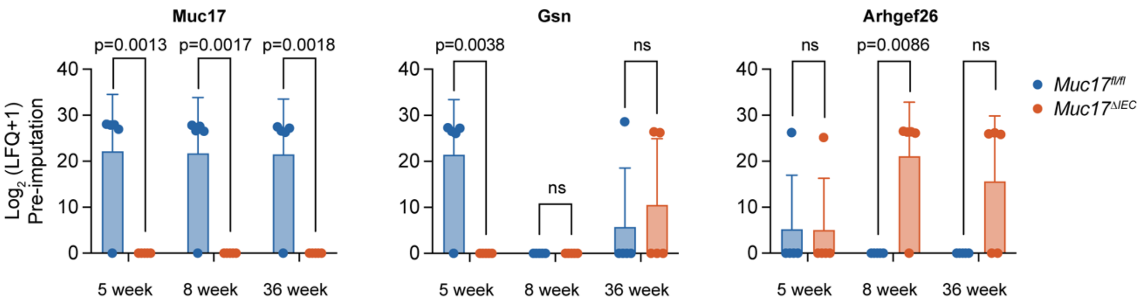

Supplemental Figure 6. Longitudinal proteomic analysis of isolated jejunal epithelial cells from *Muc17*$^{fl/fl}$ and *Muc17*$^{\Delta IEC}$ mice.

Abundance of selected proteins in 5-, 8-, and 36-week-old *Muc17*$^{fl/fl}$ and *Muc17*$^{\Delta IEC}$ mice. Significance was determined by two-way ANOVA followed by Šidák correction.

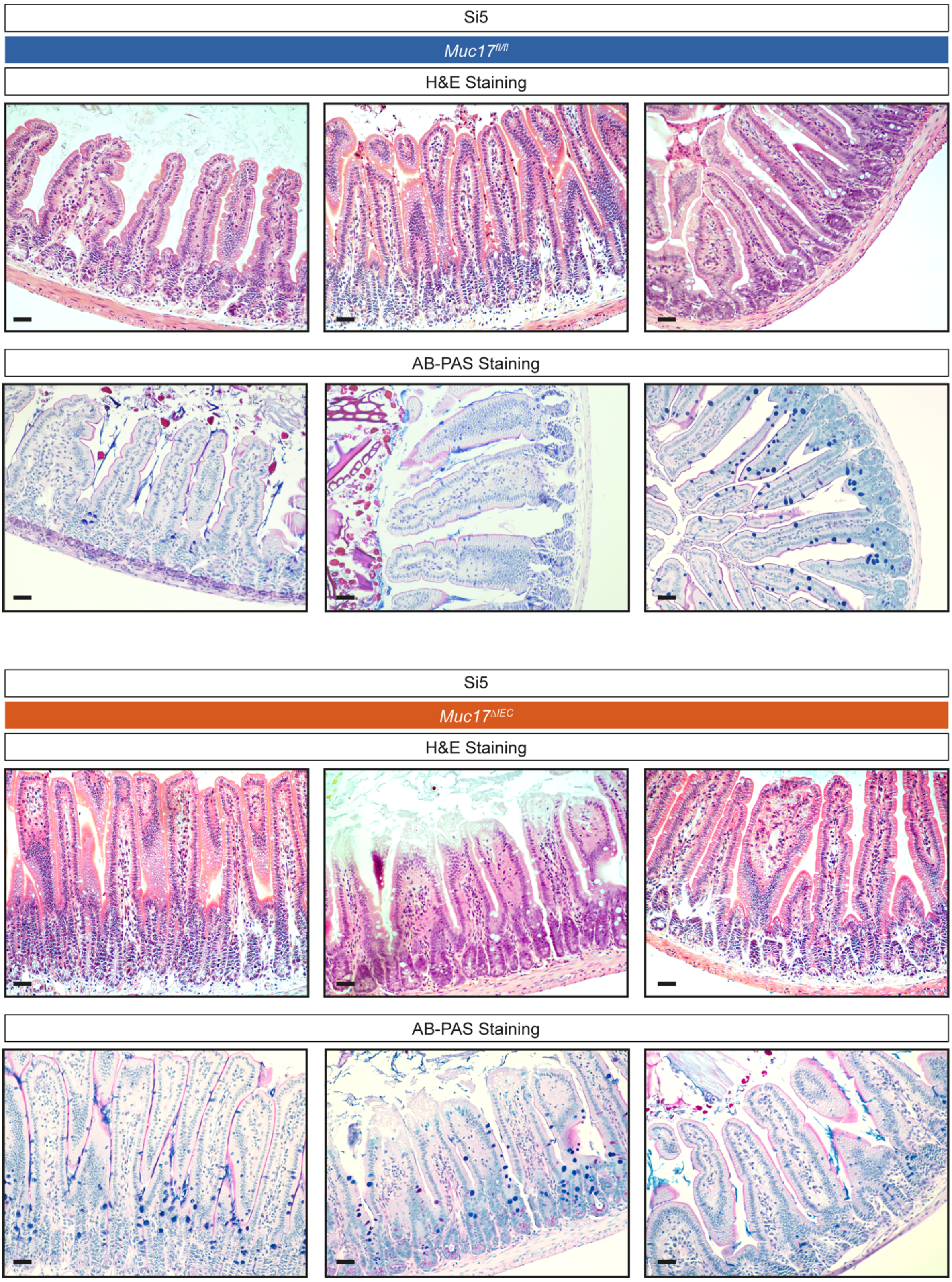

Supplemental Figure 7. Morphology of jejunum of *Muc17$^{fl/fl}$* and *Muc17$^{\Delta IEC}$* mice.

Hematoxylin-eosin and AB-PAS staining of jejunal section of 36-week-old *Muc17$^{fl/fl}$* and *Muc17$^{\Delta IEC}$* mice. Scale bar 50 µm.

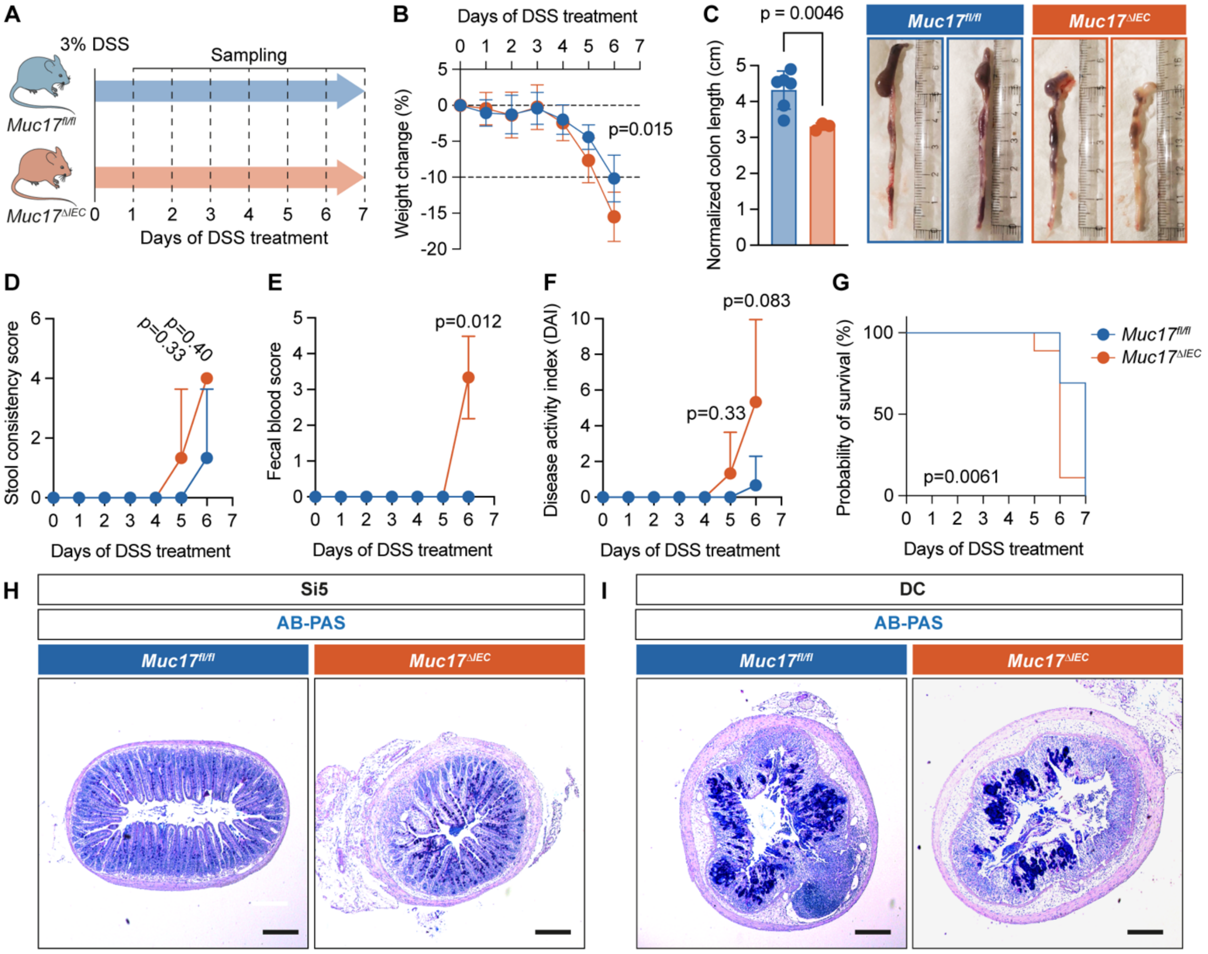

Supplemental Figure 8. Muc17 does not protect against experimental colitis.

(**A**) Schematic illustration of the DSS challenge protocol and sampling time points. (**B**) Weight loss of DSS-treated *Muc17*$^{fl/fl}$ and *Muc17*$^{\Delta IEC}$ mice, depicted as percent of the initial weight at the start of the challenge (time point 0 days). n=13 for *Muc17*$^{fl/fl}$, n=9 for *Muc17*$^{\Delta IEC}$. (**C**) Weight-normalized colon length. n=6 for *Muc17*$^{fl/fl}$, n=3 for *Muc17*$^{\Delta IEC}$. Representative images of colonic segments from *Muc17*$^{fl/fl}$ and *Muc17*$^{\Delta IEC}$ mice exposed to the DSS challenge. (**D**) Stool consistency score of *Muc17*$^{fl/fl}$ and *Muc17*$^{\Delta IEC}$ mice after the DSS challenge. n=6 for *Muc17*$^{fl/fl}$, n=3 for *Muc17*$^{\Delta IEC}$. (**E**) Fecal blood score of *Muc17*$^{fl/fl}$ and *Muc17*$^{\Delta IEC}$ mice after DSS challenge. n=6 for *Muc17*$^{fl/fl}$, n=3 for *Muc17*$^{\Delta IEC}$. (**F**) Disease activity index of *Muc17*$^{fl/fl}$ and *Muc17*$^{\Delta IEC}$ mice after DSS challenge. n=6 for *Muc17*$^{fl/fl}$, n=3 for *Muc17*$^{\Delta IEC}$. (**G**) Probability of survival of *Muc17*$^{fl/fl}$ and *Muc17*$^{\Delta IEC}$ mice exposed to DSS challenge. n=13 for *Muc17*$^{fl/fl}$, n=9 for *Muc17*$^{\Delta IEC}$. (**H**) Histological sections of *Muc17*$^{fl/fl}$ and

Muc17$^{\Delta IEC}$ jejunum (Si5) after DSS treatment stained with AP-PAS. Scale bar 100 µm. (**I**) Histological sections of *Muc17*$^{fl/fl}$ and *Muc17*$^{\Delta IEC}$ distal colon (DC) after DSS treatment stained with AP-PAS. Scale bar 100 µm. Significance was determined by two-way ANOVA followed by Šidák correction (B), Mann-Whitney test (C-F), and Log-rank (Mantel-Cox) test (G).

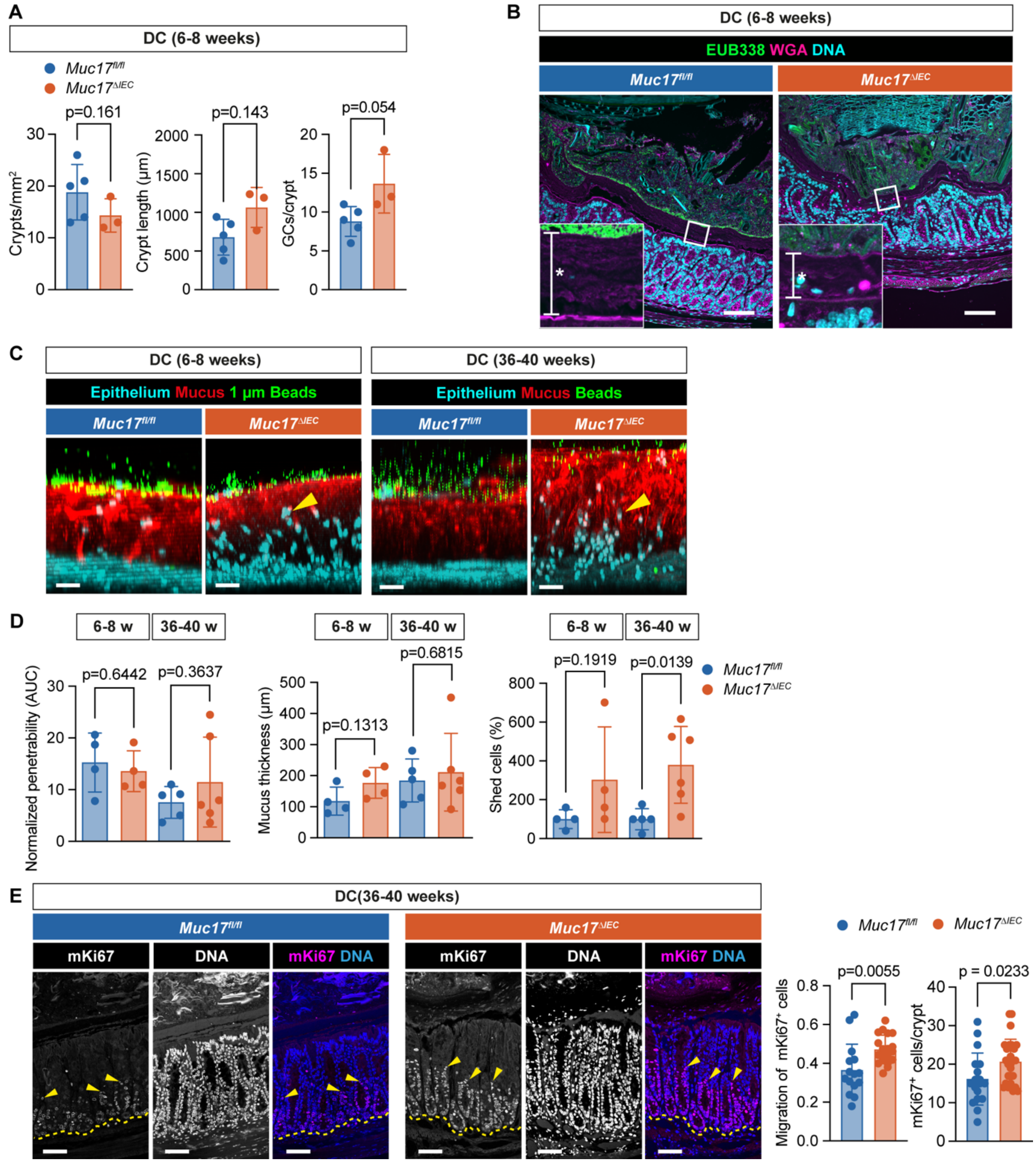

Supplemental Figure 9. Age-dependent mucus barrier defects in the distal colon of *Muc17*$^{\Delta IEC}$ mice housed under baseline SPF conditions.

(**A**) Quantification of the number of crypts per mm^2 of histological section, crypt length, and goblet cells (GCs) per crypt in the distal colon (DC) of *Muc17*$^{fl/fl}$ and *Muc17*$^{\Delta IEC}$ mice. n=6 for *Muc17*$^{fl/fl}$, n=3 for *Muc17*$^{\Delta IEC}$. (**B**) Confocal micrographs of *Muc17*$^{fl/fl}$ and *Muc17*$^{\Delta IEC}$ DC stained for bacteria (EUB338, green), epithelium and mucus (WGA, magenta), and DNA

(cyan). Asterisks mark the inner mucus layer (IML). Scale bar 50 µm. (**C**) Representative images of *ex vivo* mucus penetrability assay in 6–8- and 36–40-week-old *Muc17*$^{fl/fl}$ and *Muc17*$^{\Delta IEC}$ DC. DNA (epithelium, cyan), mucus (red), and 1 µm FluoSpheres microbeads (green). Yellow arrow points to shed epithelial cells. Scale bar 30 µm. (**D**) Quantification of mucus penetrability, mucus thickness, and shed epithelial cells in 6–8- and 36–40-week-old *Muc17*$^{fl/fl}$ and *Muc17*$^{\Delta IEC}$ DC. n=4-6 per group. (**E**) Immunohistochemistry of mKi67 (magenta) and DNA (blue) in 36–40-week-old *Muc17*$^{fl/fl}$ and *Muc17*$^{\Delta IEC}$ DC. Each channel is shown in grayscale. Yellow arrows point to mKi67^{+} cells with maximum migration along the crypt axis. The dashed yellow line depicts the crypt bottom. Scale bar 50 µm. Quantification of the migration of mKi67^{+} cells along the crypts and absolute numbers of mKi67^{+} cells in 36–40-week-old *Muc17*$^{fl/fl}$ and *Muc17*$^{\Delta IEC}$ DC. n=5-12 crypts per mouse, 3 mice per group. Significance was determined by Mann-Whitney test (A) and unpaired t-test (D-E).

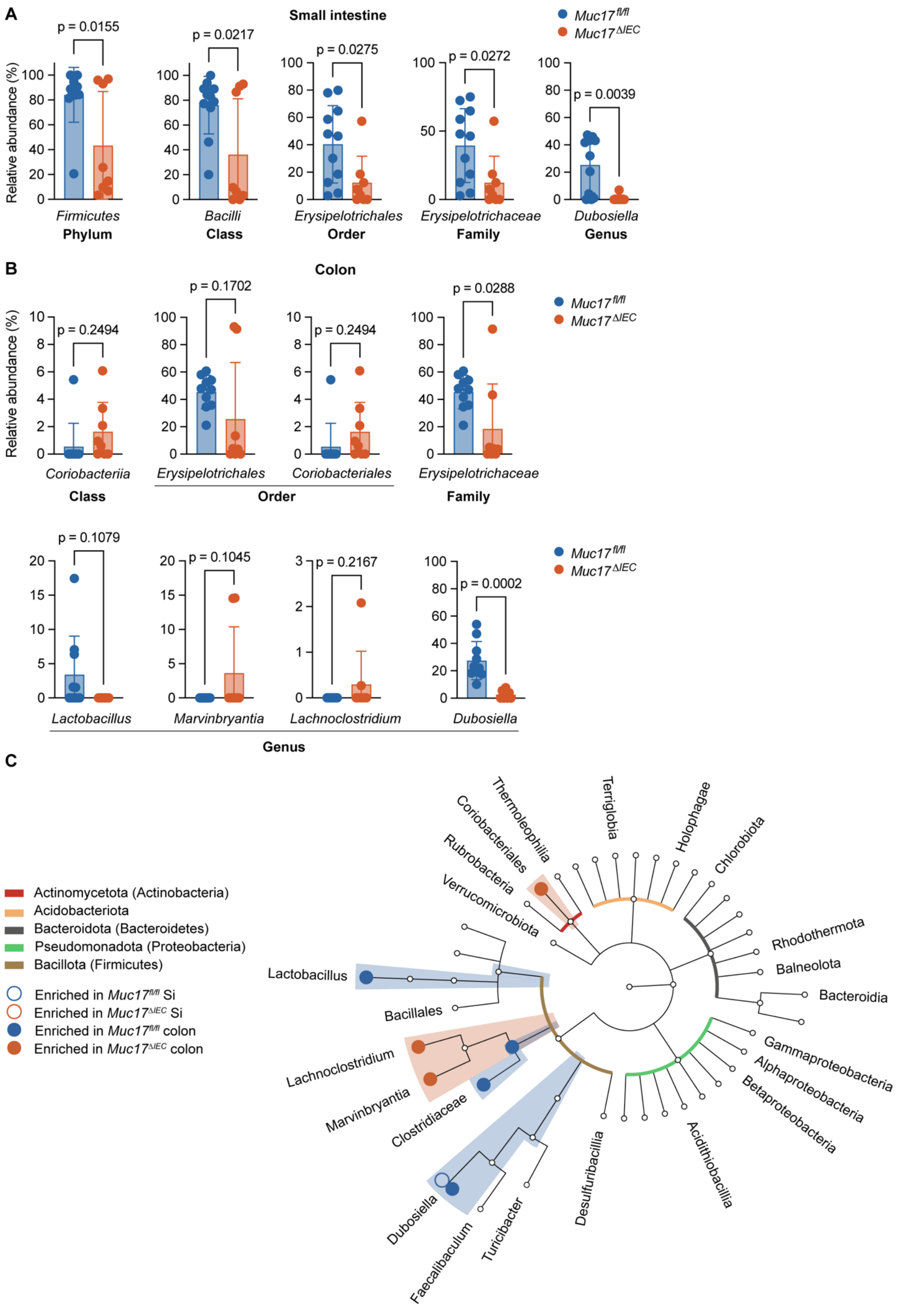

Supplemental Figure 10. Relative abundances of bacterial taxa enriched in *Muc17*$^{fl/fl}$

and *Muc17*$^{\Delta IEC}$ mice. (A) Relative abundance of enriched taxa in the small intestine of *Muc17*$^{fl/fl}$ and *Muc17*$^{\Delta IEC}$ mice. **(B)** Relative abundance of enriched taxa in the colon of *Muc17*$^{fl/fl}$ and *Muc17*$^{\Delta IEC}$ mice. Significance was determined using unpaired t-test. **(C)** Phylogenetic tree displaying bacterial taxa that are enriched in the small intestine (Si) and colon of *Muc17*$^{fl/fl}$ and *Muc17*$^{\Delta IEC}$ mice.

Supplemental Table 1. Clinical metadata and sample information. (separate file)

Supplemental Table 2. Longitudinal proteomic profiling of Si5 IECs in *Muc17*$^{fl/fl}$ and *Muc17*$^{\Delta IEC}$ mice. (separate file)

Supplemental Table 3. Primer sequences for 16S V3/V4 amplicon library preparation for luminal microbiota profiling and Index sequences for Adapterama scheme. (separate file)

Movie 1. Time-lapse *ex vivo* glycocalyx permeability assay in non-inflamed ileal biopsies from non-IBD and CD patients.

Representative movies of biopsy explants, stained for the brush border (CellMask, white) and incubated with *E. coli*$^{GFP+}$ (green). Yellow arrows indicate points of contact between *E. coli*$^{GFP+}$ and the brush border. Scale bar 10 μm.
