# Supplementary material for: MUC17 is an essential small intestinal glycocalyx component that is disrupted in Crohn’s disease
Source: JCI Insight. 2024 Dec 19;10(3):e181481. doi: 10.1172/jci.insight.181481 (PMC11948581; doi:10.1172/jci.insight.181481)
Supplement: Unedited blot and gel images [file jciinsight-10-181481-s020.pdf]

Unedited blot and gel images

MUC17 is an essential small intestinal glycocalyx component that is disrupted in Crohn's disease

Elena Layunta *et al.*

*Corresponding author. Email thaher.pelaseyed@medkem.gu.se

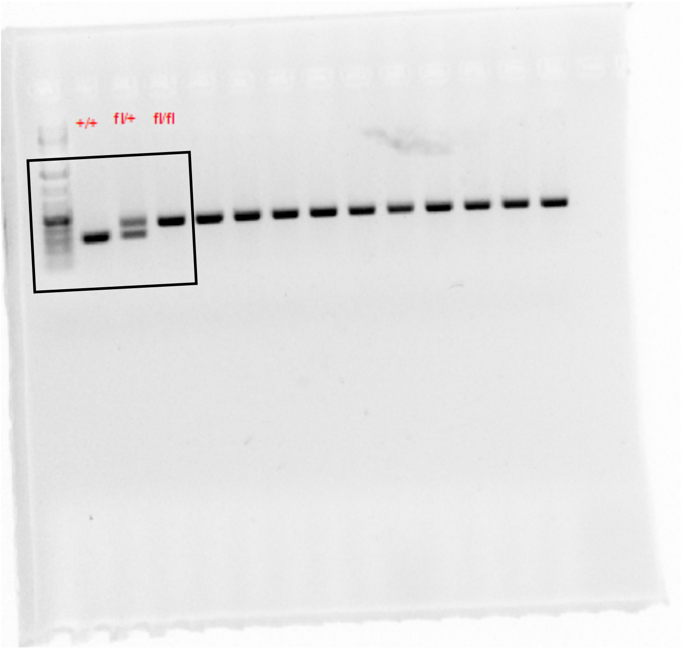

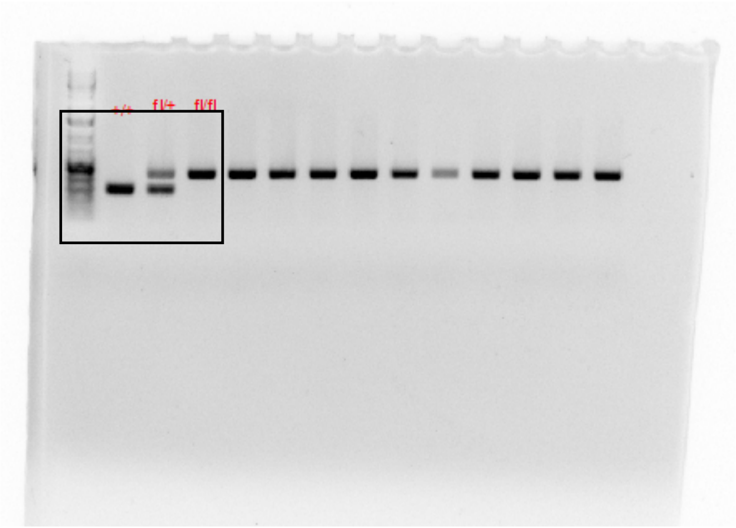

Figure S2B. Representative DNA agarose gels showing genotyping of the floxed *Muc17* allele. M, molecular weight.

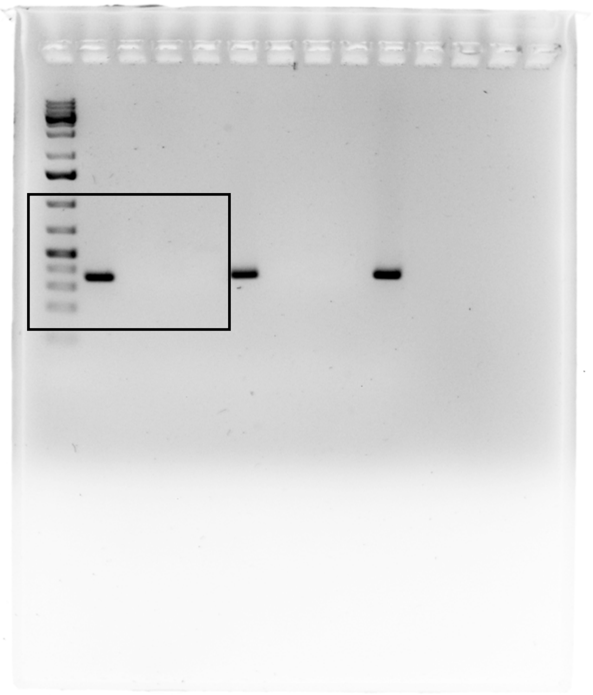

Figure S2C. Representative DNA agarose gels showing genotyping of the floxed *Muc17* alleles in the presence and absence of the *Vil1-Cre* allele in the small intestine (Si) and muscle tissues. M, molecular weight.

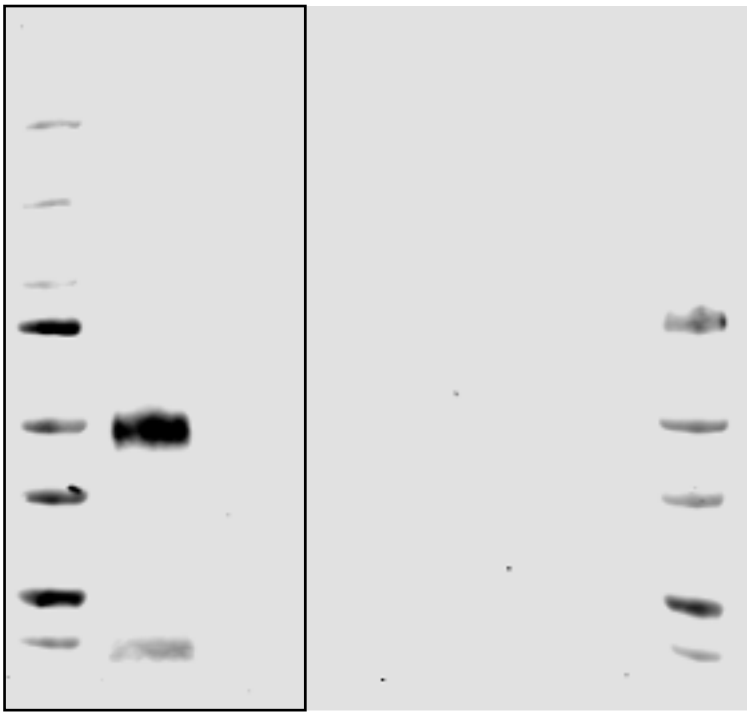

Figure S2D. Immunoblot (IB) of luminal vesicles isolated from the small intestine of *Muc17*$^{fl/fl}$ and *Muc17*$^{\Delta IEC}$ mice.
